# Prostanoid signaling in retinal cells elicits inflammatory responses relevant to early-stage diabetic retinopathy

**DOI:** 10.1186/s12974-024-03319-w

**Published:** 2024-12-23

**Authors:** Amy K. Stark, John S. Penn

**Affiliations:** 1 Department of Pharmacology, Vanderbilt University, Nashville, TN USA; 2 Department of Ophthalmology and Visual Sciences, Vanderbilt University Medical Center, Nashville, TN USA

## Abstract

Inflammation is a critical driver of the early stages of diabetic retinopathy (DR) and offers an opportunity for therapeutic intervention before irreversible damage and vision loss associated with later stages of DR ensue. Nonsteroidal anti-inflammatory drugs (NSAIDs) have shown mixed efficacy in slowing early DR progression, notably including severe adverse side effects likely due to their nonselective inhibition of all downstream signaling intermediates. In this study, we investigated the role of prostanoids, the downstream signaling lipids whose production is inhibited by NSAIDs, in promoting inflammation relevant to early-stage DR in two human retinal cell types: Müller glia and retinal microvascular endothelial cells. When cultured in multiple conditions modeling distinct aspects of systemic diabetes, Müller glia significantly increased production of prostaglandin E_2_ (PGE_2_), whereas retinal endothelial cells significantly increased production of prostaglandin F_2α_ (PGF_2α_). Müller glia stimulated with PGE_2_ or PGF_2α_ increased proinflammatory cytokine levels dose-dependently. These effects were blocked by selective antagonists to the EP2 receptor of PGE_2_ or the FP receptor of PGF_2α_, respectively. In contrast, only PGF_2α_ stimulated adhesion molecule expression in retinal endothelial cells and leukocyte adhesion to cultured endothelial monolayers, effects that were fully prevented by FP receptor antagonist treatment. Together these results identify PGE_2_-EP2 and PGF_2α_-FP signaling as novel, selective targets for future studies and therapeutic development to mitigate or prevent retinal inflammation characteristic of early-stage DR.

**Supplementary Information:**

The online version contains supplementary material available at 10.1186/s12974-024-03319-w.

## Introduction

Diabetic retinopathy (DR), a neurovascular complication of diabetes mellitus, is a leading cause of irreversible vision loss in working-age adults in America and worldwide [1–3]. Clinically, DR presents in two phases: early-stage nonproliferative diabetic retinopathy (NPDR) and late-stage proliferative diabetic retinopathy (PDR) [4–6]. NPDR is characterized by vascular pathologies including vessel hyperpermeability, pericyte death, capillary occlusion and atrophy, basement membrane thickening, and clinically observable retinal microaneurysms [6, 7]. Concurrently, degeneration of neurons, particularly retinal ganglion cells and photoreceptors, and the consequent decline in synaptic functioning and neurovascular coupling also occur [7–9]. Additionally, a rising inflammatory response occurs in the retina early in disease progression, presumably in reaction to conditions of systemic diabetes and the resulting tissue damage [6, 10]. The transition from NPDR to PDR is marked by the onset of retinal neovascularization, the abnormal angiogenic growth of blood vessels in response to increasing vascular and tissue damage and consequent retinal ischemia [5, 6]. Neovascularization in PDR is the primary cause of irreversible vision loss occurring in DR [6, 7].

Currently, intraocular anti-vascular endothelial growth factor (VEGF) injection to inhibit hyperpermeability and neovascularization serves as the standard of care for DR [11]. However, these anti-VEGF drugs—the only approved therapies for DR—are used to treat later-stages of DR, when irreparable retinal damage is likely to have already occurred. There is a pressing need to investigate therapies for DR that intervene at earlier stages of disease before severe damage ensues, and inflammation in NPDR offers one such target of intervention.

Within the retina, numerous cell types play distinct roles in the regulation of tissue health and visual function. Of particular note are Müller glia and retinal microvascular endothelial cells, two key cell types involved in regulating retinal responses to conditions of diabetes and the consequent inflammatory damage occurring in NPDR. Müller glia are eye-specific glial support cells that span nearly the full thickness of the retina. These cells play critical roles in supporting normal functions of other retinal cell types through maintenance of the blood-retina barrier, metabolic control and nutrient supply, and uptake and recycling of ions and neurotransmitters [6, 12, 13]. Further, Müller glia respond to damaging stimuli in the retina, such as conditions of diabetes, by elevating production of cytokines and chemokines that can stimulate further activation of inflammatory cascades in other retinal cells [6, 7, 12, 13]. In the context of DR, among the most critical cell types the Müller glia affect are the retinal microvascular endothelial cells, which form the luminal walls of retinal capillaries. Inflammatory damage to these cells can promote further cytokine production, blood-retina barrier breakdown, apoptosis, and the adhesion of circulating leukocytes to the retinal endothelium, known as leukostasis [6, 14]. As leukostasis progresses, it can lead to capillary occlusion and focal retinal ischemia, hallmarks of advancing DR [6]. Dysregulation of cytokine levels in Müller glia and leukostasis markers in retinal endothelial cells can be probed in vitro to analyze the critical inflammatory responses of each cell type that may promote the initial stages of NPDR.

Nonsteroidal anti-inflammatory drugs (NSAIDs) are well-established medications to reduce pain and inflammation by preventing the metabolism of arachidonic acid by cyclooxygenase-1 (COX-1) and COX-2 enzymes [15]. The potential of COX inhibition to treat DR was first identified in a corelative analysis of patients taking salicylates to manage rheumatoid arthritis, which showed that diabetic patients in the cohort demonstrated slowed DR progression [16]. Subsequently, systemic, intravitreal, and topical uses of NSAIDs were investigated as therapeutic strategies for DR prevention in several clinical trials with varying results [17]. For example, trials of high doses of systemic aspirin or sulindac showed decreased DR progression over the durations of these studies [18, 19]. In contrast, another trial with a lower dose of aspirin revealed no benefit for DR [20]. Further, a trial of systemic celecoxib (COX-2 selective) for DR was terminated early due to risk for severe cardiovascular side effects with no significant retinal benefit observed during the truncated study [21]. The chronic, systemic use of NSAIDs has been shown to promote severe cardiovascular, cerebrovascular, gastrointestinal, and/or renal side effects, among others [22]. Additional trials have tested intravitreal or topical NSAID drugs for DR or diabetic macular edema, a complication that can occur at any stage of DR, but these therapies similarly did not show significant effects on disease progression [23, 24]. Overall, clinical trials of COX inhibition by NSAIDs to manage DR progression have yielded inconsistent findings with a number showing no therapeutic benefit.

More selective targeting of the COX metabolism pathway could provide a more efficacious and reliable option. In this pathway, arachidonic acid is converted by COX-1 or COX-2 into unstable intermediates that are rapidly converted by specific synthase enzymes into the five prostanoids: prostaglandins PGD_2_, PGE_2_, PGF_2α_, PGI_2_, and thromboxane TXA_2_ [25]. These distinct lipids signal with specificity via nine G protein-coupled receptors (GPCRs), which are DP1 and DP2 for PGD_2_; EP1, EP2, EP3, and EP4 for PGE_2_; FP for PGF_2α_, IP for PGI_2_, and TP for TXA_2_ [26]. Furthermore, the primary Gα subtype coupling varies among these GPCRs for additional differentiation of cellular and molecular effects downstream. Receptors DP1, EP2, EP4, and IP couple primarily to Gα_s_ to activate adenylyl cyclase to produce cAMP. DP2 and EP3 couple to Gα_i_ to inhibit adenylyl cyclase and prevent cAMP production. EP1, FP, and TP couple to Gα_q_ to activate phospholipase C and ultimately elevate intracellular calcium levels [26]. The roles of prostanoids and their receptors have been a subject of basic and clinical research in DR as well as several other retinal vascular diseases [27].

Based on the potential therapeutic benefits for DR patients demonstrated in some—but not all—clinical trials of NSAIDs, we hypothesize that antagonism of individual prostanoid receptors might prove efficacious in limiting inflammation relevant to early-stage DR without the adverse effects caused by broad-spectrum COX inhibition by NSAIDs. To test this, we employed cell culture models using primary human Müller glia (hMG) and primary human retinal microvascular endothelial cells (hRMEC). We cultured each cell type under three conditions that model aspects of systemic diabetes to measure the secretion levels of each of the five prostanoids and determine which were altered. We then assayed the dose–response effects of altered prostanoids on NPDR-relevant cell behaviors and determined the receptors mediating each of these effects. Our goal was to identify *selective* anti-inflammatory therapeutic targets for early-stage DR intervention.

## Methods

### Primary human retinal cell culture

Primary human Müller glia (hMG) were isolated from human donor eyes obtained within 24 h postmortem from the National Disease Research Interchange using a protocol adapted from Hicks and Courtois [28]. Briefly, the retina was dissected and dissociated in low glucose (1 g/L) Dulbecco's Modified Eagle Medium (DMEM; Gibco; Grand Island, NY) containing trypsin and collagenase to select for Müller glia survival and proliferation. hMG were maintained in DMEM supplemented with 10% fetal bovine serum (FBS; R&D Systems; Minneapolis, MN) and 1% penicillin/streptomycin (Gibco) in a cell culture incubator held at 37 °C, 5% CO_2_, and 95% humidity. Passage 5 and 6 cells from multiple human donors were used for all experiments.

Primary human retinal microvascular endothelial cells (hRMEC) were obtained from Cell Systems (Kirkland, WA). Cells were grown in culture dishes coated in Attachment Factor (Cell Systems) and maintained in endothelial basal medium (EBM; Cell Systems) supplemented with 10% FBS and Endothelial Cell Growth Medium SingleQuots (Lonza; Basel, Switzerland) in a cell culture incubator held at 37 °C, 5% CO_2_, and 95% humidity. Passage 7 and 8 cells were used for all experiments.

### Treatment of retinal cells

Treatment of hMG began when cells reached 90% confluence. Media were changed from 10 to 2% FBS DMEM + penicillin/streptomycin for 12 h prior to the start of treatment. Where applicable, hMG were pretreated with prostanoid receptor antagonists SC-51322 (100 nM–1 μM; Cayman Chemical; Ann Arbor, MI), PF-04418948 (100 nM–1 μM; Cayman Chemical), DG-041 (100 nM–1 μM; Tocris; Bristol, United Kingdom), L-161,982 (100 nM–1 μM; Cayman Chemical), AL8810 (100 nM–10 μM; Cayman Chemical), or DMSO vehicles for 1 h in fresh 2% FBS DMEM + penicillin/streptomycin. For treatments, hMG were stimulated with recombinant human IL-1β (1 ng/mL in water; Sino Biological; Beijing, China), palmitic acid (250 μM in DPBS with 1% bovine serum albumin; Sigma-Aldrich; St. Louis, MO), D-glucose (24.5 mM; Sigma-Aldrich), l-glucose (24.5 mM; Sigma-Aldrich), PGE_2_ (1 nM–10 μM in DMSO; Cayman Chemical), or PGF_2α_ (1 nM–10 μM in DMSO; Cayman Chemical) with proper vehicles in fresh 2% FBS DMEM + penicillin/streptomycin for times specified in each experiment.

Treatment of hRMEC began when cells reached 90% confluence. For mass spectrometry experiments, media were changed from 10 to 5% FBS EBM + SingleQuots 12 h prior to the start of treatment, then cells were stimulated with human IL-1β (1 ng/mL), palmitic acid (250 μM), d-glucose (24.5 mM), or l-glucose (24.5 mM) with relevant vehicles in 5% FBS EBM + SingleQuots for 24 h. For prostanoid stimulation experiments, where applicable, hRMEC were pretreated with FP receptor antagonist AL8810 (100 nM–10 μM) or DMSO vehicle for 30 min in fresh 10% FBS EBM + SingleQuots. For treatments, hRMEC were stimulated with PGE_2_ (1 nM–10 μM) or PGF_2α_ (1 nM–10 μM) with DMSO vehicles in fresh 10% FBS EBM + SingleQuots for times specified in each experiment.

### Liquid chromatography-tandem mass spectrometry (LC–MS/MS) of secreted prostanoids

After treatment, media were harvested for mass spectrometry of secreted prostanoids, and total protein from adherent cells was collected in RIPA buffer (Sigma-Aldrich). LC–MS/MS was performed by the Eicosanoid Core Laboratory at Vanderbilt University. Media samples were spiked with a mix of deuterated standards including PGD_2_, PGE_2_, PGF_2α_, 6-keto-PGF_1α_ (stable metabolite of PGI_2_), and TXB_2_ (stable metabolite of TXA_2_) dissolved in 25% methanol in water. Samples were vortexed and centrifuged at 10,000 × g for 10 min to pellet protein, then supernatants were extracted on an Oasis MAX uElution plate (Waters Corp.; Milford, MA), washed with methanol followed by 25% methanol in water, and eluted with 50/50 acetonitrile/2-propanol containing 5% formic acid to an elution plate. Samples were run on a Waters Xevo TQ-XS triple quadrupole mass spectrometer connected to a Waters Acquity I-Class UPLC. Analytes were separated with gradient elution using an Acquity PFP column with a mobile phase A of 0.01% formic acid in water and a mobile phase B of acetonitrile. Samples were analyzed using fragmentation of PGD_2_ and PGE_2_ (separated chromatographically) at m/z 351, PGF_2α_ at m/z 353, 6-keto-PGF_1α_ at m/z 369, and TXB_2_ at m/z 369. Prostanoid levels were normalized to total protein measured by Pierce BCA assay (Thermo Fisher Scientific; Waltham, MA) and reported as pg secreted prostanoid/µg total protein.

### Prostaglandin ELISAs

Following treatment of hMG with glucose, palmitic acid, inflammatory cytokines, or respective vehicles for 2–96 h, media were collected and analyzed using Prostaglandin E_2_ or Prostaglandin F_2α_ Monoclonal ELISA Kits (Cayman Chemical) according to the manufacturer’s protocols. Sample concentrations were interpolated from prostaglandin standard curves using GraphPad Prism 10 software (La Jolla, CA) and reported as pg prostanoid/ml media. For the palmitic acid stimulation experiment, data were analyzed using a simple linear regression on GraphPad Prism 10.

### Proteome profiler cytokine array

hMG were stimulated with 1 μM PGE_2_ or DMSO vehicle for 6 h, then conditioned media were assayed with a Proteome Profiler Human XL Cytokine Array Kit (R&D Systems) according to the manufacturer’s protocol. Membrane pairs (vehicle- and PGE_2_-treated) were imaged simultaneously using an Amersham Imager 600 chemiluminescent reader (GE Healthcare; Chicago, IL). Mean gray values of technical duplicates were recorded using Fiji/ImageJ (National Institutes of Health; Bethesda, MD) for analysis. Background levels were subtracted and mean gray values of image pairs were scaled by ratios of 1:1:3:6 to normalize data and account for differences in chemiluminescent exposure of independent experiments.

### qRT-PCR

After treatment, cells were lysed, RNA was isolated using the RNeasy Mini Kit (Qiagen; Hilden, Germany), and cDNA was synthesized using the High-Capacity cDNA Reverse Transcription Kit (Applied Biosystems; Waltham, MA). qRT-PCR was performed using a StepOnePlus Real-Time PCR System (Applied Biosystems) with TaqMan Universal PCR Master Mix (Applied Biosystems) and TaqMan probes as follows: *IL6* (Hs00985639_m1), *CXCL8* (Hs00174103_m1), *IL1B* (Hs01555410_m1), *ICAM1* (Hs00164932_m1), *VCAM1* (Hs01003372_m1), *SELE* (Hs00174057_m1), *PTGDR* (Hs00235003_m1), *PTGDR2* (Hs00173717_m1), *PTGER1* (Hs00168752_m1), *PTGER2* (Hs00168754_m1), *PTGER3* (Hs00168755_m1), *PTGER4* (Hs00168761_m1), *PTGFR* (Hs00168763_m1), *PTGIR* (Hs00168765_m1), *TXA2R* (Hs00169054_m1), *TBP* (Hs00427620_m1). Gene expression fold change was normalized relative to *TBP* gene expression, which was unchanged in all experimental conditions.

### Cytokine ELISAs

Following treatment, hMG culture media were assayed using ProQuantum human IL-6, IL-8, and IL-1β Immunoassay ELISA kits (Invitrogen; Carlsbad, CA) according to the manufacturer’s protocol. Sample concentrations were interpolated from cytokine standard curves using GraphPad Prism 10 software and reported as pg cytokine/ml media.

### cAMP ELISAs

hMG were cultured in 96-well plates to 90% confluence. Cells were pretreated for 1 h with PF-04418948, L-161,982, or DMSO vehicle where applicable. Cells were stimulated for 15 min with 1 nM–10 μM PGE_2_ or DMSO vehicle to promote cAMP production. cAMP levels were measured from treated samples with a cAMP Assay Colorimetric Competitive ELISA Kit (ab234585; Abcam; Cambridge, United Kingdom) according to the manufacturer’s protocol. Sample concentrations were interpolated from cAMP standard curves using GraphPad Prism 10 software and normalized to cAMP levels in vehicle-treated controls.

### Western blot

After treatment, cells were harvested in RIPA buffer (Sigma-Aldrich) containing cOmplete Mini EDTA-free Protease Inhibitor Cocktail tablets (Roche; Basel, Switzerland). Lysates were centrifuged at 10,000 × *g* for 10 min, then supernatants were isolated for analysis. Total protein concentration was measured by BCA. Equal concentrations of protein were loaded and resolved on 4–20% Mini-PROTEAN TGX polyacrylamide gels (Bio-Rad; Hercules, CA), transferred using nitrocellulose transfer stacks on the iBlot 2 system (Invitrogen), and blocked in Intercept TBS Blocking Buffer (LI-COR; Lincoln, NE). Blots were stained with primary antibodies diluted in Blocking Buffer with 0.2% Tween 20 (Sigma-Aldrich) as follows: rabbit anti-EP1 (#101740, 1:250; Cayman Chemical), rabbit anti-EP2 (#101750, 1:250; Cayman Chemical), rabbit anti-EP3 (#101760, 1:250; Cayman Chemical), rabbit anti-EP4 C-Term (#101775, 1:250; Cayman Chemical), mouse anti-ICAM-1 (sc-8439, 1:1000; Santa Cruz Biotechnology; Dallas, TX), rabbit anti-VCAM-1 (ab134047, 1:1000; Abcam), and mouse anti-β-actin (#3700, 1:1000; Cell Signaling Technology; Danvers, MA). Blots were washed four times in TBS with 0.1% Tween 20 then stained with secondary antibodies diluted in Blocking Buffer with 0.2% Tween 20 (Sigma-Aldrich) as follows: 680LT donkey anti-mouse (926-68022; 1:10000; LI-COR) and 800CW donkey anti-rabbit (926-32213, 1:10000; LI-COR). Blots were imaged on a LI-COR Odyssey CLx reader and quantified using Fiji/ImageJ. Target protein levels were normalized to β-actin and reported as fold-change versus vehicle-treated samples.

### Static adhesion

hRMEC were cultured in 24-well plates and treated in relevant conditions. Meanwhile, human peripheral blood mononuclear cells (PBMCs) obtained from Precision for Medicine (Frederick, MD) were stained with NucBlue Hoechst 33342 live cell stain (Invitrogen) for 20 min. PBMCs were pelleted and resuspended in fresh 10% serum EBM. Following hRMEC treatment with prostanoids for 6–10 h, treatment media were removed and approximately 250,000 PBMCs in 500 μl EBM were added per well. Culture plates were returned to the cell culture incubator for 30 min. Following incubation, media were aspirated to remove nonadherent PBMCs, and wells were washed gently three times with warm Dulbecco's Phosphate-Buffered Saline (DPBS; Gibco). hRMEC monolayers with adherent PBMCs were fixed with 4% paraformaldehyde (PFA; Electron Microscopy Sciences; Hatfield, PA) in DPBS for 10 min at room temperature and subsequently washed twice with DPBS. Wells were imaged by capturing a 5-field-by-5-field 10x objective stitched image in brightfield (to ensure hRMEC monolayer integrity) and DAPI filter (to quantify adherent PBMCs) on a Nikon Eclipse Ti inverted microscope. DAPI-stained PBMCs were quantified using Fiji/ImageJ. Wells with hRMEC monolayers that were not intact were excluded from quantification. PBMC counts per well were normalized to the average count of PBMCs in vehicle-treated wells. Data from four independent experiments are shown (n = 14–20 per treatment).

### Statistical analysis

**Fig. 1:**
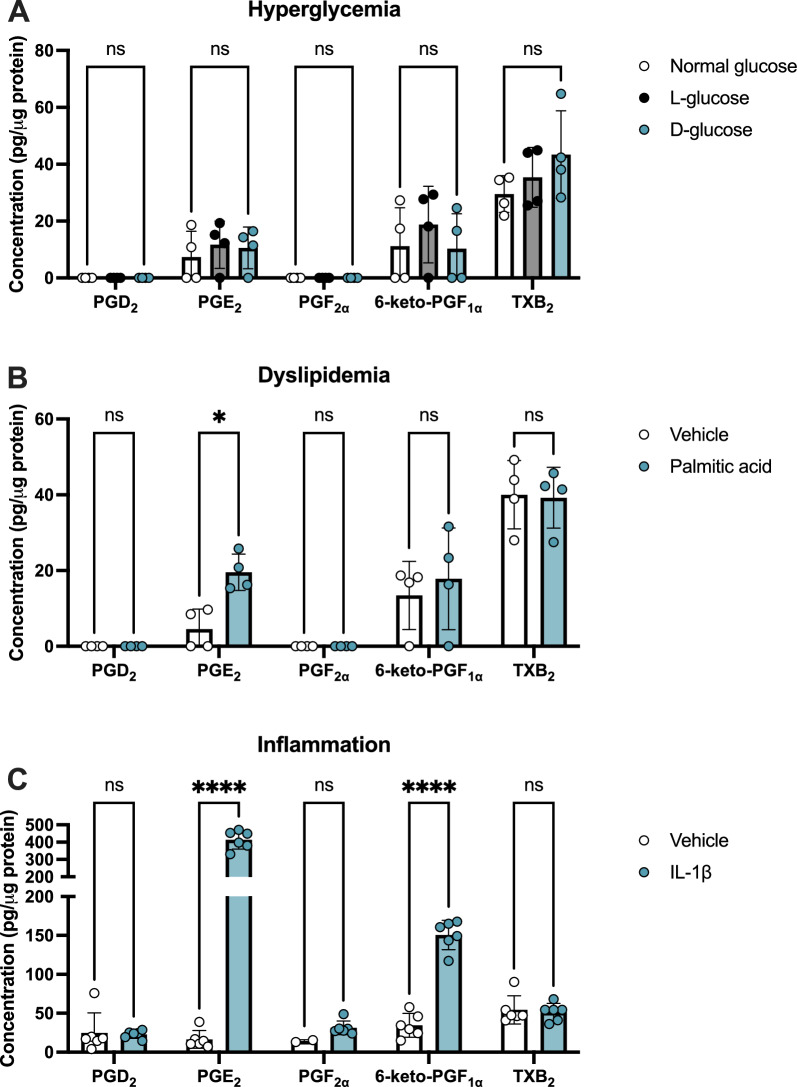
Prostanoid production by hMG in conditions simulating systemic diabetes. hMG were stimulated with (**A**) additional 24.5 mM l-glucose or d-glucose, (**B**) 250 μM palmitic acid, or (**C**) 1 ng/mL recombinant IL-1β or relevant controls for 24 h, then media were collected for LC–MS/MS targeting PGD_2_, PGE_2_, PGF_2α_, 6-keto-PGF_1α_ (PGI_2_ metabolite) and TXB_2_ (TXA_2_ metabolite). Data were normalized as pg prostanoid per μg of total protein from cell lysates (*n* = 2–6). Data represent mean ± SD. Two-way ANOVAs with Šídák post-hoc tests were used. Statistically significant differences are represented as *P < 0.05, ****P < 0.0001, ns (not significant) *P* > 0.05

**Fig. 2:**
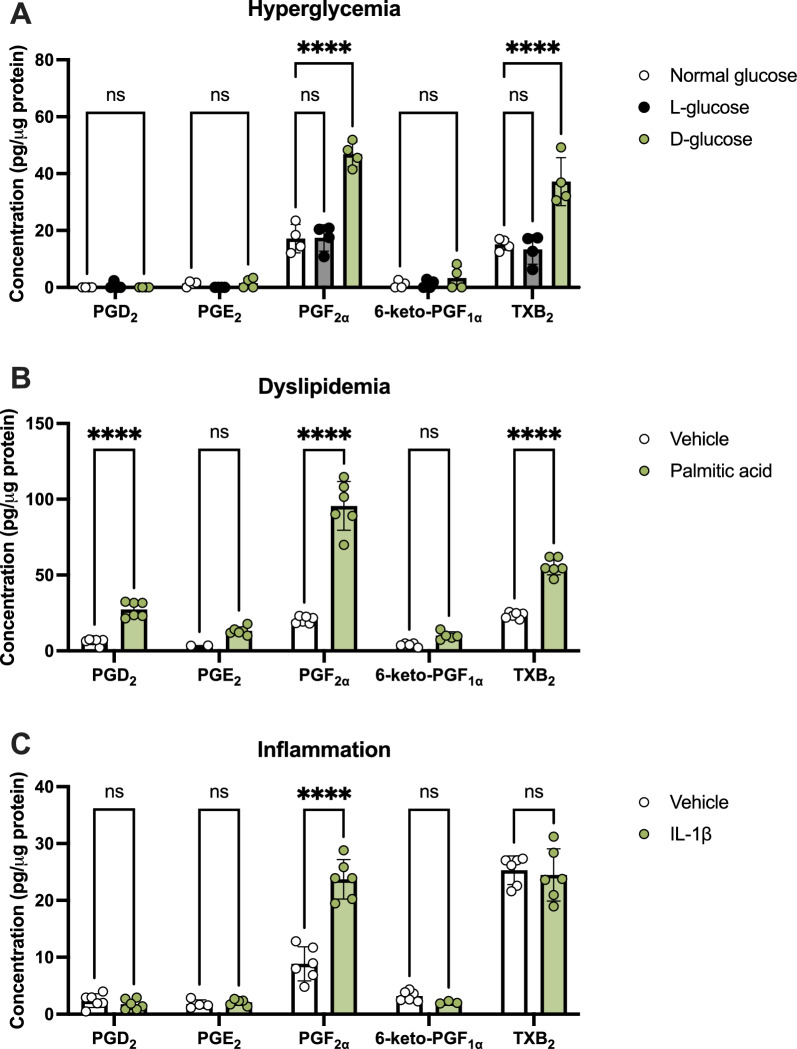
Prostanoid production from hRMEC in conditions simulating systemic diabetes. hRMEC were stimulated with (**A**) 24.5 mM l-glucose or d-glucose, (**B**) 250 μM palmitic acid, or (**C**) 1 ng/mL recombinant IL-1β or relevant controls for 24 h, then media were collected for LC–MS/MS targeting PGD_2_, PGE_2_, PGF_2α_, 6-keto-PGF_1α_ (PGI_2_ metabolite) and TXB_2_ (TXA_2_ metabolite). Data were normalized as pg prostanoid per μg of total protein from cell lysates (n = 2–6). Data represent mean ± SD. Two-way ANOVAs with Šídák post-hoc tests were used. Statistically significant differences are represented as ****P < 0.0001, ns (not significant) *P* > 0.05

**Fig. 3:**
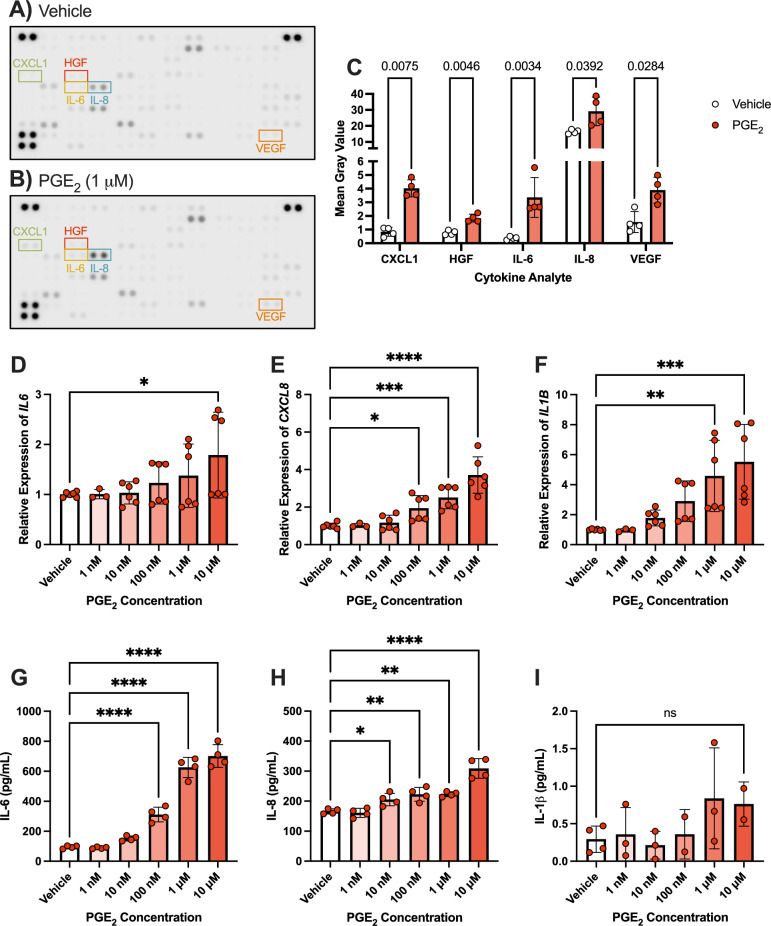
PGE_2_ stimulates elevation of proinflammatory cytokine levels. Representative cytokine arrays treated with hMG-conditioned media after 6 h of stimulation with (**A**) vehicle or (**B**) 1 μM PGE_2_. (**C**) Significantly altered targets averaged from all arrays (n = 4). Multiple ratio paired T tests with Holm-Šídák post-hoc tests were used for 3C and adjusted *P* values are shown. (**D)** *IL6*, (**E**) *CXCL8*, and (**F**) *IL1B* qRT-PCR gene expression changes in hMG stimulated with vehicle or elevating PGE_2_ concentrations for 6 h (n = 3–6). (**G**) IL-6, (**H**) IL-8, and (**I**) IL-1β ELISA protein level changes from media of hMG stimulated with vehicle or elevating PGE_2_ concentrations for 6 h (n = 2–4). Data represent mean ± SD. One-way ANOVAs with Dunnett post-hoc tests were used for 3D-I. Statistically significant differences are represented as *P < 0.05, **P < 0.01, ***P < 0.001, ****P < 0.0001, ns (not significant) *P* > 0.05

**Fig. 4:**
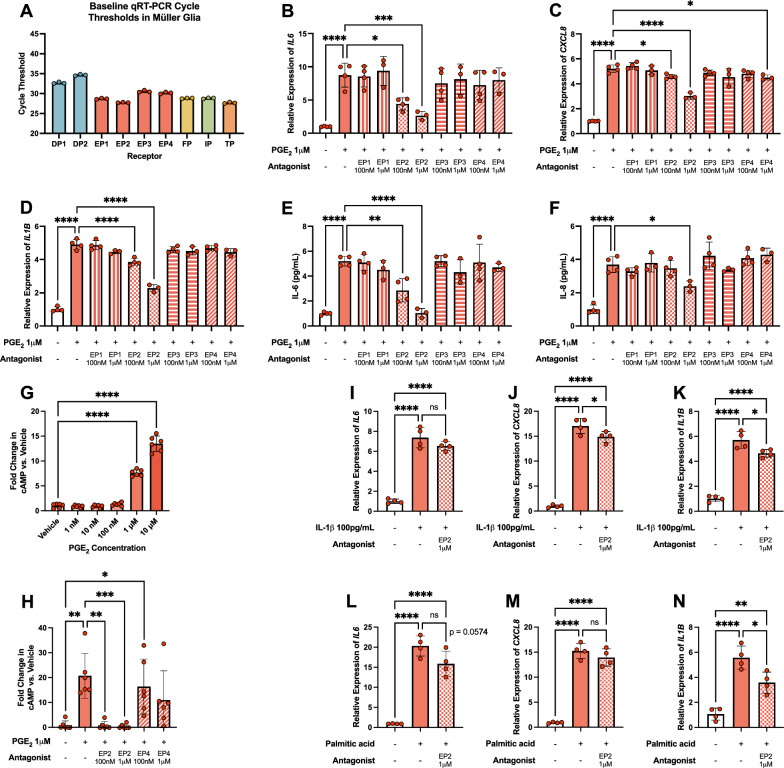
PGE_2_-EP2 signaling mediates proinflammatory cytokine production in hMG. (**A**) qRT-PCR cycle thresholds of prostanoid receptor genes in unstimulated hMG (n = 3). (**B**) *IL6* gene expression in hMG stimulated with vehicle or PGE_2_ ± prostanoid receptor antagonist for 2 h (n = 3–4). (**C**) *IL1B* and (**D**) *CXCL8* gene expression in hMG stimulated with vehicle or PGE_2_ ± prostanoid receptor antagonist for 6 h (n = 3–4). (**E**) IL-6 protein levels in culture media from hMG stimulated with vehicle or PGE_2_ ± prostanoid receptor antagonist for 6 h (n = 3–4). (**F**) IL-8 protein levels in culture media from hMG stimulated with vehicle or PGE_2_ ± prostanoid receptor antagonist for 10 h (n = 3–4). (**G**) cAMP production from hMG stimulated with vehicle or elevating PGE_2_ concentrations for 15 min (n = 6). (**H**) cAMP production from hMG stimulated with vehicle or 1 μM PGE_2_ ± EP2 or EP4 antagonists for 15 min (n = 6). (**I**) *IL6*, (**J**) *CXCL8*, and (**K**) *IL1B* gene expression in hMG stimulated with vehicle or 100 pg/mL IL-1β ± EP2 antagonist for 6 h (n = 4). (**L**) *IL6*, (**M**) *CXCL8*, and (**N**) *IL1B* gene expression in hMG stimulated with vehicle or 250 μM palmitic acid ± EP2 antagonist for 24 h (n = 4). Data represent mean ± SD. One-way ANOVAs with Dunnett post-hoc tests were used for 4B-G. One-way ANOVAs with Tukey post-hoc tests were used for 4H-N. Statistically significant differences are represented as *P < 0.05, **P < 0.01, ***P < 0.001, ****P < 0.0001, ns (not significant) *P* > 0.05

**Fig. 5:**
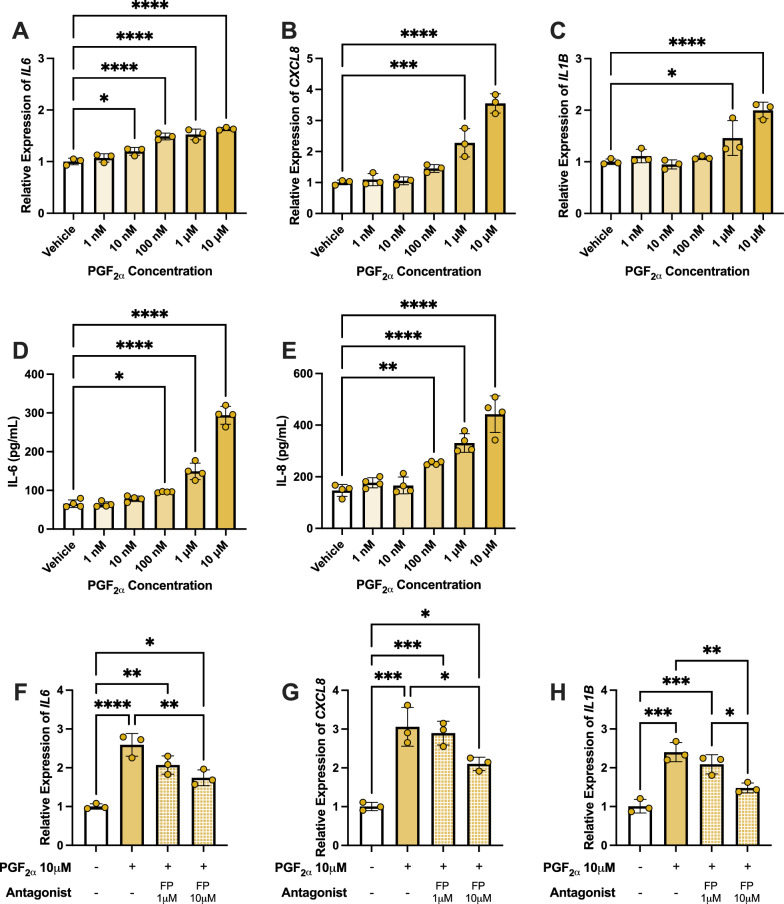
PGF_2α_-FP signaling promotes proinflammatory cytokine production in hMG. (**A**) *IL6*, (**B**) *CXCL8*, and (**C**) *IL1B* qRT-PCR gene expression changes in hMG stimulated with vehicle or elevating PGF_2α_ concentrations for 6 h (n = 3). (**D)** IL-6 and (**E**) IL-8 ELISA protein level changes from media of hMG stimulated with vehicle or elevating PGF_2α_ concentrations for 6 h (n = 4). (**F**) *IL6* gene expression in hMG stimulated with vehicle or PGF_2α_ ± FP receptor antagonist for 2 h (n = 3). (**G**) *CXCL8* and (**H**) *IL1B* gene expression in hMG stimulated with vehicle or PGF_2α_ ± FP receptor antagonist for 6 h (n = 3). Data represent mean ± SD. One-way ANOVAs with Dunnett post-hoc tests were used for 5A-E. One-way ANOVAs with Tukey post-hoc tests were used for 5F-H. Statistically significant differences are represented as *P < 0.05, **P < 0.01, ***P < 0.001, ****P < 0.0001

**Fig. 6:**
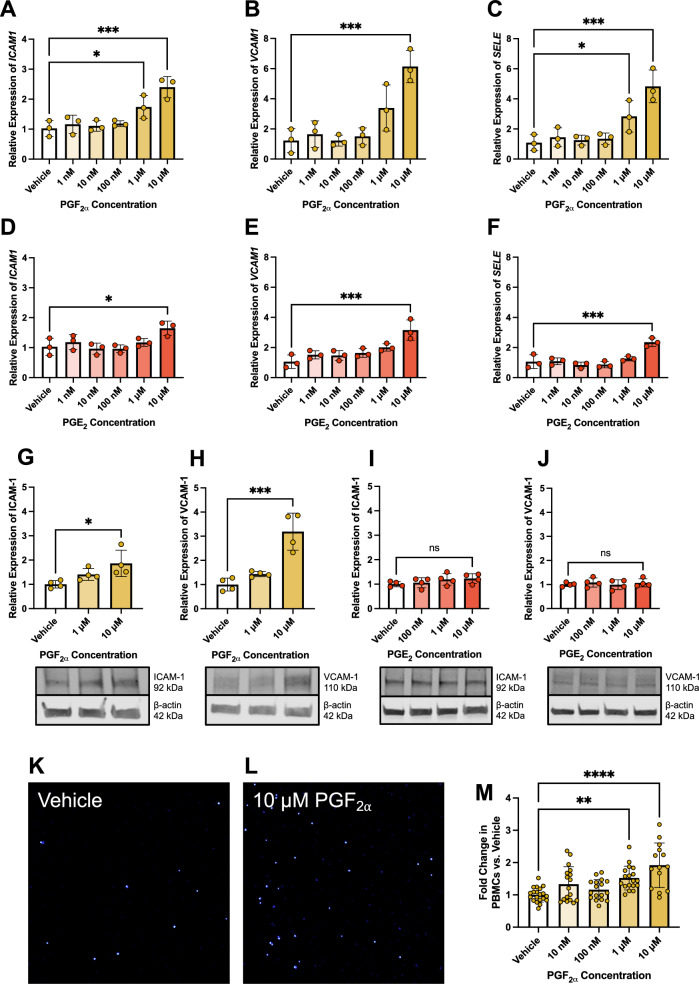
PGF_2α_, but not PGE_2_, promotes leukostasis-relevant activity at gene, protein, and cell behavior levels in hRMEC. (**A**) *ICAM1*, (**B)** *VCAM1*, and (**C**) *SELE* qRT-PCR gene expression changes in hRMEC stimulated with vehicle or elevating PGF_2α_ concentrations for 6 h (n = 3). (**D**) *ICAM1*, (**E**) *VCAM1*, and (**F**) *SELE* qRT-PCR gene expression changes in hRMEC stimulated with vehicle or elevating PGE_2_ concentrations for 6 h (n = 3). (**G**) ICAM-1 and (**H**) VCAM-1 western blot protein levels and representative blots from hRMEC stimulated with vehicle or elevating PGF_2α_ concentrations for 6 h (n = 4). (**I**) ICAM-1 and (**J**) VCAM-1 protein levels and representative western blots from hRMEC stimulated with vehicle or elevating PGE_2_ concentrations for 6 h (n = 4). Representative images of static PBMC adhesion after (**K**) vehicle or (**L**) 10 μM PGF_2α_ stimulation for 6 h. (**M**) Static adhesion results with vehicle or elevating PGF_2α_ concentrations for 6 h (n = 14–20). Data represent mean ± SD. One-way ANOVAs with Dunnett post-hoc tests were used. Statistically significant differences are represented as *P < 0.05, **P < 0.01, ***P < 0.001, ****P < 0.0001, ns (not significant) *P* > 0.05

**Fig. 7:**
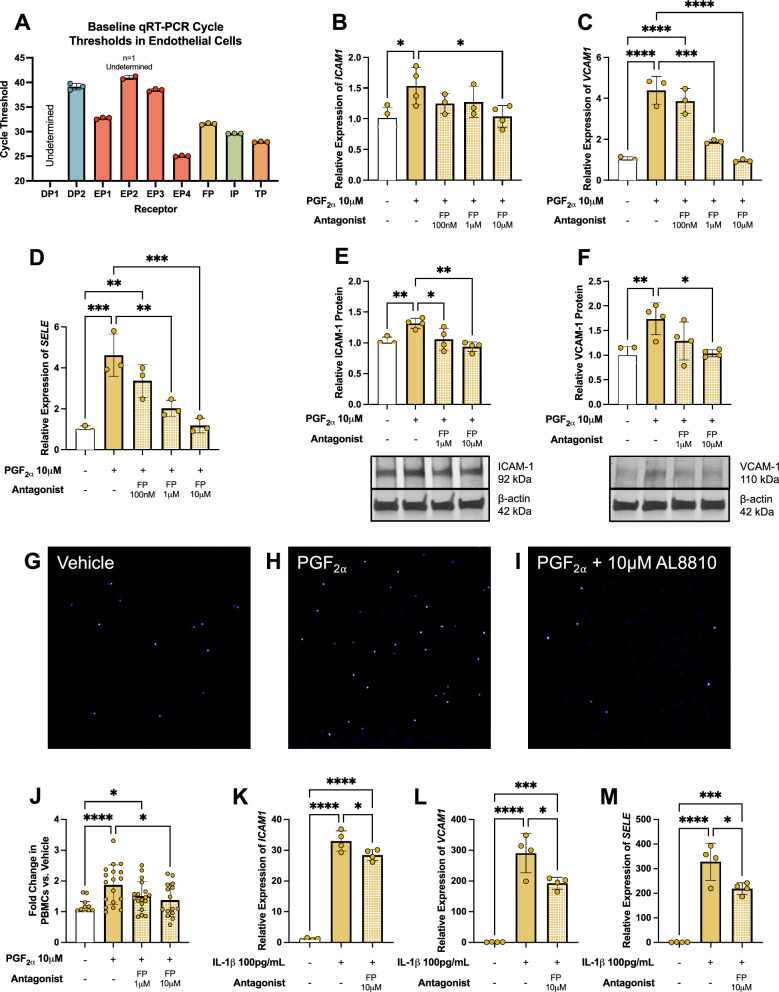
PGF_2α_-FP signaling mediates leukocyte adhesion in hRMEC. (**A**) qRT-PCR cycle thresholds of prostanoid receptor genes in unstimulated hRMEC (n = 3). (**B**) *ICAM1*, (**C**) *VCAM1*, and (**D**) *SELE* gene expression in hRMEC stimulated with vehicle or PGF_2α_ ± FP receptor antagonist for 6 h (n = 3). (**E**) ICAM-1 and (**F**) VCAM-1 western blot protein levels and representative blots from hRMEC stimulated with vehicle or PGF_2α_ ± FP receptor antagonist for 10 h (n = 4). Representative images of static PBMC adhesion after (**G**) vehicle, (**H**) 10 μM PGF_2α_, or (**I**) 10 μM PGF_2α_ + 10 μM FP receptor antagonist treatment. (**J**) Static adhesion results with vehicle or PGF_2α_ ± FP receptor antagonist for 10 h (n = 15–18). (**K**) *ICAM1*, (**L**) *VCAM1*, and (**M**) *SELE* gene expression in hRMEC stimulated with vehicle or 100 pg/mL IL-1β ± FP antagonist for 6 h (n = 4). Data represent mean ± SD. One-way ANOVAs with Tukey post-hoc tests were used. Statistically significant differences are represented as *P < 0.05, **P < 0.01, ***P < 0.001, ****P < 0.0001

Data analysis was performed using GraphPad Prism 10. Data are represented as mean ± standard deviation (SD). Normality was assessed using Shapiro–Wilk tests with a significance level of 0.05 before applying parametric analyses. Two-way ANOVAs with Šídák post-hoc multiple comparison tests were used for LC–MS/MS experiments with two independent variables (treatment and prostanoid) in Figs. 1 and 2. Multiple ratio paired T tests with Holm-Šídák post-hoc multiple comparison tests were used for the cytokine array experiment in Fig. 3C. One-way ANOVAs with Dunnett (to compare to one relevant treatment group; ex: Fig. 5A–E) or Tukey (to compare all treatment groups; ex: Fig. 5F–H) post-hoc multiple comparison tests were used for experiments with one independent variable in Figs. 3–7. The threshold for significance was *P* < 0.05.

## Results

### hMG produce PGE_2_ in conditions simulating systemic diabetes

As diabetes affects the body systemically to lead to DR onset and progression, we aimed to characterize the effects of multiple systemic changes occurring in diabetes that may alter the production of prostanoids within the eye. We first analyzed these responses in primary human Müller glia (hMG), cells essential for the initiation and propagation of retinal inflammation in response to disease. Here, hMG were cultured for 24 h in media supplemented to model conditions of hyperglycemia, dyslipidemia, and chronic inflammation occurring in diabetes, and prostanoid levels were measured by LC–MS/MS. In all three experiments, there was significant variation attributable to the prostanoid target as an independent variable, which indicates differences in the baseline prostanoid levels in addition to any effects of treatment. First, hyperglycemia was modeled by supplementation of normal 5.5 mM D-glucose DMEM media, which represents the upper range of fasting plasma glucose levels of nondiabetic patients [29], with an additional 24.5 mM D-glucose, which models fasting plasma glucose levels of severe diabetes, or 24.5 mM L-glucose as an osmotic control. Elevated D-glucose supplementation caused no significant changes in any prostanoid levels when compared to normal media or L-glucose supplemented media (Fig. 1A). ELISAs targeted to PGE_2_ and PGF_2α_ confirmed that glucose supplementation did not affect these levels relative to normal glucose controls for up to 96 h of treatment (*supplemental *Fig. 1A, B), indicating that hyperglycemia is not a major contributor to prostanoid production by hMG. Second, dyslipidemia was modeled by supplementing media with 250 μM palmitic acid—the concentration of this free fatty acid in the bloodstreams of patients with type 2 diabetes [30]—and compared with vehicle supplementation. Palmitic acid stimulation resulted in a 4.30-fold elevation of PGE_2_, whereas other prostanoid levels were unchanged (Fig. 1B). This elevation of PGE_2_ exhibited a linear trend over time, beginning with significant elevation after 4 h and maintained through 48 h of palmitic acid stimulation (*supplemental *Fig. 1C). Third, chronic inflammation resulting from systemic diabetes was modeled by the acute addition of proinflammatory cytokines to media. At equal concentrations of 1 ng/mL, the proinflammatory cytokine IL-1β, which is elevated in the serum and vitreous humor of patients with DR [31, 32], promoted the strongest elevation of prostanoid production in human Muller glia compared with TNFα, another cytokine also elevated in DR patient serum and vitreous humor, or lipopolysaccharide (LPS), an endotoxin found in gram-negative bacteria that serves as an inflammatory stimulus not related to diabetes (*supplemental *Fig. 1D). Here, IL-1β significantly elevated PGE_2_ by 25.1-fold and 6-keto-PGF_1α_, a stable metabolite of PGI_2_, by 4.36-fold (Fig. 1C). Overall, conditions of hyperglycemia did not yield any changes in prostanoid production by hMG, but both dyslipidemia and inflammation resulted in elevated PGE_2_ levels in these cells.

### hRMEC produce PGF_2α_ in conditions simulating systemic diabetes

Because the retina is composed of a wide variety of cell types each with distinct roles, we hypothesized that different cell types may produce and respond to prostanoids in discrete ways; therefore, we also studied the effects of systemic diabetes conditions on prostanoid production in hRMEC. As in hMG, hRMEC were cultured for 24 h in media supplemented with elevated glucose for hyperglycemia, palmitic acid for dyslipidemia, and IL-1β for chronic inflammation. Subsequently, prostanoid levels were measured by LC–MS/MS. Two-way ANOVAs showed that each independent variable (treatment or prostanoid target) as well as the interaction between them was a source of significant variability for hyperglycemia, dyslipidemia, and inflammation experiments. Unlike hMG, hRMEC responded to hyperglycemic conditions with a 2.74-fold elevation of PGF_2α_ and a 2.46-fold elevation of TXB_2_, a stable metabolite of TXA_2_, in high D-glucose conditions relative to unsupplemented media controls (Fig. 2A). L-glucose supplementation as an osmotic control showed no significant change in any target versus unsupplemented media, indicating the effects observed by D-glucose stimulation are due to a hyperglycemic effect rather than an osmotic effect. In high palmitic acid conditions modeling dyslipidemia, hRMEC exhibited significant elevations of PGF_2α_ by 4.67-fold, PGD_2_ by 4.36-fold, and TXB_2_ by 2.37-fold (Fig. 2B). Finally, when treated with IL-1β as a model of chronic inflammation, only elevation of PGF_2α_ by 2.68-fold in hRMEC was observed, whereas other prostanoids were not significantly changed relative to vehicle (Fig. 2C). Together, these data show an elevation of PGF_2α_ most consistently in hRMEC cultured under conditions modeling diabetes.

### PGE_2_ stimulates proinflammatory cytokine expression in hMG

Based on the most potent and consistent production of PGE_2_ from hMG in response to conditions of systemic diabetes, we sought to investigate the autocrine effects of this elevated prostanoid on cytokine production by hMG, which could drive retinal inflammation key to early DR progression. To model these effects, hMG were stimulated with 1 μM PGE_2_ or vehicle for 6 h, and conditioned media were tested in a Proteome Profiler cytokine array of human cytokine and chemokine responses. Arrays suggest upregulation of numerous targets relevant broadly to inflammatory and/or angiogenic responses due to PGE_2_ stimulation (Fig. 3A–C). Five targets were significantly elevated by PGE_2_ in four independent experiments: CXCL1 (GROα), hepatocyte growth factor (HGF), IL-6, IL-8, and VEGF (Fig. 3C).

In studying these responses of hMG to putative autocrine PGE_2_ signaling further, we validated the effects of elevated PGE_2_ on gene and protein levels of the DR-relevant targets IL-6 and IL-8, which have been well-characterized in DR pathogenesis [32–34], as well as IL-1β, which stimulated strong prostanoid production in Figs. 1C and 2C and is also known to drive DR progression [31, 32]. The effects of PGE_2_ in promoting proangiogenic VEGF production in mouse Müller glia has been previously published by our laboratory [35], so this response was not reinvestigated here. hMG showed an elevation of *IL6*, *CXCL8* (IL-8), and *IL1B* gene expression when stimulated with increasing concentrations of PGE_2_ for 6 h. Target gene expression was normalized to *TBP* gene expression, which was unchanged in all experiments, (Fig. 3D–F). Additionally, cytokine ELISAs showed significantly elevated protein levels of IL-6 and IL-8 in the media of cells after 6 h of stimulation with PGE_2_ concentrations (Fig. 3G, H). However, despite robust effects of PGE_2_ on *IL1B* gene expression, ELISAs yielded extremely low concentrations of IL-1β protein in both control and PGE_2_-treated samples, not significantly different from each other (Fig. 3I).

### PGE_2_-induced cytokine elevation in hMG is mediated by the EP2 receptor

PGE_2_ signals with high affinity via four GPCRs with different downstream Gα subunit coupling. With these distinct downstream signaling pathways, determining the EP receptor(s) by which PGE_2_ signals to elevate cytokine expression in hMG is important to identify therapeutic targets. hMG express all four EP receptors as determined by raw qRT-PCR cycle threshold (Ct) values for each EP receptor gene in unstimulated cells, where a lower Ct represents a higher baseline expression (Fig. 4A). Furthermore, EP1-4 protein levels were also detected by western blot in unstimulated hMG cultures (supplemental Fig. S2).

Here, hMG were pretreated for 1 h with vehicle or 100 nM–1 μM of a selective antagonist to each EP receptor: SC-51322 for EP1, PF-04418948 for EP2, DG-041 for EP3, or L-161,982 for EP4. Subsequently, 1 μM PGE_2_ was added to stimulate cytokine production.

Cytokine gene expression was evaluated after two timepoints of PGE_2_ stimulation—2 h and 6 h—to optimally assess peak expression of individual targets, which could differ from the representative-yet-isolated timepoint assessed in Fig. 3. Stimulation of hMG for longer times did not further elevate gene expression levels (*supplemental *Fig. 3). *IL6* expression was maximally elevated after 2 h of PGE_2_ stimulation, and only the EP2 antagonist PF-04418948 significantly reduced *IL6* expression (Fig. 4B; *supplemental *Fig. 4A, B). *CXCL8* and *IL1B* expression were maximally elevated after 6 h of stimulation; similarly, only PF-04418948 decreased *IL1B* and *CXCL8* expression (Fig. 4C, D; *supplemental *Fig. 4C). A high concentration of the EP4 antagonist L-161,982 also caused a small, yet significant, decrease in *CXCL8* expression after 6 h, likely due to off-target effects (Fig. 4D). No other EP receptor antagonist decreased PGE_2_-induced gene expression, suggesting that these proinflammatory effects are driven by the EP2 receptor.

Secreted cytokine levels of IL-6 and IL-8 were analyzed by ELISA after 6 h and 10 h of PGE_2_ stimulation, optimized for peak timing. After 6 h, IL-6 was significantly elevated in culture medium by PGE_2_, and only PF-04418948 treatment inhibited IL-6 production and secretion (Fig. 4E; *supplemental *Fig. 4D). After 10 h, IL-8 was maximally elevated by PGE_2_ and IL-6 remained elevated, while only PF-04418948 inhibited cytokine production and secretion (Fig. 4F; *supplemental *Fig. 4E).

Downstream GPCR activation was assayed by hMG production of cAMP, the downstream effector of Gα_s_-coupled GPCRs including EP2 and EP4. cAMP levels were dose-dependently elevated in hMG with increasing PGE_2_ concentrations after 15 min of stimulation (Fig. 4G). Additionally, pretreatment with 100 nM or 1 μM of PF-04418948 for 1 h fully inhibited the elevation of cAMP induced by 1 μM PGE_2_, whereas 100 nM or 1 μM of the EP4 antagonist L-161,982 had no effect on PGE_2_-stimulated cAMP levels (Fig. 4H). Together, these results support a role for the EP2 receptor in mediating the proinflammatory effects of PGE_2_ in hMG.

Finally, the capacity for EP2 antagonism to prevent cytokine elevation by diabetes-relevant conditions was modeled by stimulating hMG with IL-1β or palmitic acid, stimuli that promoted PGE_2_ production in Fig. 1, in the presence or absence of PF-04418948. hMG were pretreated with 100 nM PF-04418948 or vehicle for 1 h followed by stimulation. At 100 pg/mL, IL-1β significantly elevated *IL6*, *CXCL8*, and *IL1B* gene expression after 6 h of stimulation, and PF-04418948 significantly inhibited *CXCL8* induction by 12.8% and *IL1B* induction by 18.6% (Fig. 4I–K). However, *IL6* induction was not significantly inhibited by PF-04418948 in these conditions (Fig. 4I). Similarly, 250 μM palmitic acid promoted *IL6*, *CXCL8*, and *IL1B* expression after 24 h of stimulation, and PF-04418948 significantly inhibited *IL1B* induction by 35.6% and inhibited *IL6* induction by 21.7%, though this was not statistically significant (p = 0.0574) (Fig. 4L–N). Here, *CXCL8* expression was not inhibited by antagonist treatment (Fig. 4M). These experiments link PGE_2_-EP2 signaling more directly to the inflammatory activation of hMG under conditions modeling aspects of systemic diabetes, yet the partial reductions with antagonist treatment enforce the notion that there are many distinct proinflammatory pathways active in such conditions in addition to the effects of PGE_2_.

### Paracrine-like signaling of PGF_2α_ via the FP receptor promotes proinflammatory cytokine production in hMG

Retinal cells reside in close proximity in vivo, so paracrine signaling through secreted molecules may critically impact the disease processes within the eye. Therefore, select paracrine roles were assayed in vitro by modeling the effects of PGF_2α_, the highest upregulated prostanoid observed by LC–MS/MS in hRMEC, on hMG cytokine production. Here, hMG were stimulated with increasing concentrations of PGF_2α_ for a single representative period of 6 h as in Fig. 3. Gene expression of *IL6*, *CXCL8*, and *IL1B* (Fig. 5A–C) and protein levels of IL-6 and IL-8 (Fig. 5D, E) were increased by PGF_2α_ dose-dependently, similarly to what was observed for the presumed autocrine signaling of PGE_2_ in these cells.

PGF_2α_ signals with specificity for a single Gα_q_-coupled receptor, the FP receptor, which is also expressed in hMG (Fig. 4A). In order to determine if these effects of PGF_2α_ were specific this cognate receptor, cytokine gene expression was assayed using the FP receptor antagonist AL8810, which has reported K_i_ values ranging from 2.6 μM to 5.7 μM in various human ocular cell types [36–38].

hMG were pretreated with AL8810 for 1 h followed by stimulation with 10 μM PGF_2α_ for 2 h or 6 h, optimized for the time of peak gene expression of the individual gene targets as in Fig. 4. After 2 h of PGF_2α_ stimulation, *IL6* gene expression was maximally induced and 10 μM AL8810 significantly inhibited the effects of PGF_2α_ (Fig. 5F). Gene expression of *CXCL8* and *IL1B* were also induced at lower levels by PGF_2α_ after 2 h of stimulation (*supplemental *Fig. 5A, B). After 6 h of PGF_2α_ stimulation, both *CXCL8* and *IL1B* were maximally expressed, prevented in each case by AL8810 (Fig. 5G, H). *IL6* expression after PGF_2α_ stimulation was not maximally expressed at this timepoint (*supplemental *Fig. 5C). Collectively, these results suggest that PGF_2α_, which may be derived from retinal microvascular endothelial cells in situ, might elicit a paracrine response from Müller glia by promoting cytokine production via the FP receptor.

### PGF_2α_, but not PGE_2_, promotes adhesion of leukocytes to hRMEC

Inflammation occurring in DR can promote dysfunction throughout the retina, notably including the adhesion of circulating leukocytes to the endothelium, known as leukostasis. This can be modeled experimentally in hRMEC by studying the gene and protein levels of key adhesion molecules, including E-selectin, which mediates the initial capture of leukocytes by the endothelium, as well as ICAM-1 and VCAM-1, which promote firm anchoring of leukocytes to the capillary walls [39]. Additionally, this behavior may be modeled in vitro by static adhesion assays, comparing the adhesion of human peripheral blood mononuclear cells (PBMCs) added to treated or untreated hRMEC monolayers.

To investigate the putative autocrine and paracrine effects of prostanoids on leukostasis outcomes, we stimulated hRMEC with PGF_2α_, induced in hRMEC under diabetic conditions to model autocrine signaling, or PGE_2_, induced in hMG under such conditions to model paracrine signaling. PGF_2α_ promoted an elevation of *ICAM1*, *VCAM1*, and *SELE* (E-selectin) gene expression after 6 h of stimulation with increasing prostaglandin stimulation (Fig. 6A–C). Additionally, PGF_2α_ stimulated ICAM-1 and VCAM-1 protein expression in these conditions (Fig. 6G, H). Notably, PGE_2_ only promoted adhesion gene expression at the highest 10 μM concentrations and at levels that were only 49–68% of the effects of PGF_2α_ on these genes (Fig. 6D–F). Furthermore, PGE_2_ failed to induce adhesion protein expression at any concentration (Fig. 6I–J), suggesting that paracrine PGE_2_ signaling does not promote leukostasis-relevant behaviors in hRMEC. In static adhesion assays to model leukostasis in vitro, PGF_2α_ dose-dependently promoted PBMC adhesion to hRMEC monolayers at 1 μM and higher concentrations (Fig. 6K–M), strengthening our notions about the role of autocrine PGF_2α_ signaling in hRMEC-leukocyte adhesion.

### Leukocyte adhesion in hRMEC is mediated by the FP receptor of PGF_2α_

PGF_2α_ signaling was analyzed at the primary receptor for this prostanoid, the FP receptor, which is expressed in hRMEC as determined by baseline qRT-PCR cycle threshold values of unstimulated cells (Fig. 7A). Pharmacologic inhibition assays were performed using the FP-selective antagonist AL8810. Here, hRMEC were pretreated with vehicle or AL8810 at 100 nM-10 μM for 30 min, then 10 μM PGF_2α_ was added to stimulate adhesion. After stimulation for 6 h, *SELE*, *ICAM1*, and *VCAM1* gene expression levels in hRMEC were decreased dose-dependently by AL8810 pretreatment down to vehicle-treated levels (Fig. 7B–D). Further, AL8810 pretreatment decreased ICAM-1 and VCAM-1 protein levels in hRMEC to vehicle-treated levels after 10 h of PGF_2α_ or vehicle stimulation (Fig. 7E, F). Finally, AL8810 dose-dependently prevented PBMC adhesion to hRMEC in a static adhesion assay under stimulation by PGF_2α_ for 10 h (Fig. 7G–J).

To evaluate the extent to which PGF_2α_-FP signaling may be responsible for the induction of leukocyte adhesion in hRMEC, cells were stimulated in the diabetes-relevant conditions modeling hyperglycemia, dyslipidemia, and chronic inflammation, each of which elevated PGF_2α_ production in Fig. 2, in the presence or absence of AL8810. hRMEC were pretreated with 10 μM AL8810 or vehicle for 30 min followed by stimulation. After 6 h of stimulation with 100 pg/mL IL-1β, expression of *ICAM1*, *VCAM1*, and *SELE* were each significantly elevated, and AL8810 significantly inhibited expression by 13.8%, 33.5%, and 33.3%, respectively (Fig. 7K–M). Stimulation with 250 μM palmitic acid for 24 h also significantly promoted expression of all three targets, but AL8810 failed to reduce their expression (*supplemental *Fig. 6A–C). Lastly, culturing cells with elevated D-glucose only modestly elevated target gene expression, significant only for *ICAM1*, and the difference with AL8810 treatment was not significant for any target (*supplemental *Fig. 6D–F). Together, these results, like the collateral experiments for PGE_2_-EP2 in hMG in Fig. 4**,** provide a partial link between PGF_2α_-FP signaling and direct inflammatory activation of hRMEC in conditions modeling aspects of diabetes. However, the differential efficacy of AL8810 against these three stimuli underscores the complex, multifactorial inflammatory processes involved in these cells.

## Discussion

Our findings show that prostanoid signaling has discrete proinflammatory roles in gene, protein, and cell behavior assays relevant to the early inflammatory stage of diabetic retinopathy. Previous basic and clinical research has targeted COX-1 and/or COX-2 inhibition by NSAIDs as a therapeutic strategy to limit DR progression due to the noted anti-inflammatory benefits of these drugs. However, the mixed successes observed in these trials prompted our effort to identify more selective targets in the COX/prostanoid signaling pathways. Using experimental approaches of relevance to early DR, our findings suggest that dysregulation of only select prostanoids, rather than all five prostanoids as targeted by NSAIDs, promotes inflammatory retinal pathologies. Therapeutic modulation of single receptors could inhibit pathogenic prostanoid signaling while leaving non-pathogenic prostanoid signaling pathways unaltered. We hypothesize that this may be important for a variety of other essential tissue responses in normal and disease conditions.

PGE_2_ was significantly elevated in cultures of hMG under conditions modeling systemic dyslipidemia and inflammation that are experienced by patients with diabetes mellitus but not under conditions modeling hyperglycemia. This result aligns with clinical findings that PGE_2_ levels were 53% higher in the vitreous humor of patients with PDR compared to nondiabetic control vitreous samples [40]. In contrast, PGF_2α_ was the only prostanoid produced at elevated levels in cultures of hRMEC after stimulation with conditions relevant to hyperglycemia, dyslipidemia, and inflammation, each of the three conditions of systemic diabetes tested. This effect is also substantiated by clinical results. One study found the PGF_2α_ metabolite 13,14-dihydro-PGF_2α_ was 178% higher in PDR patient vitreous samples compared to nondiabetic controls [41]. Similarly, serum levels of the PGF_2α_ metabolite 15-keto-dihydro-PGF_2⍺_ were significantly higher in PDR patients compared with either NPDR patients or diabetic patients with no DR [42]. These clinical findings support our hypothesis that individual prostanoid levels and/or the synthase enzymes driving prostanoid production may be dysregulated in DR, yet their differences also add complexity to the cell-type specific changes observed in our experiments. The different prostanoid production profiles from hMG and hRMEC stimulated to model systemic diabetes may reflect the distinct roles these two retinal cell types play in initiating, propagating, and sustaining retinal inflammation in DR.

Müller glia are critical regulators of the retina’s response to damaging stimuli, necessary to help maintain homeostasis and promote healthy retinal function [12]. The wide-ranging roles of these cells in sustaining the retinal environment—including neurotransmitter release, ion buffering, blood flow regulation, and cell metabolism support—indicate that normal Müller glia function is essential to retinal homeostasis [7, 12]. Activation of these cells by disease-relevant conditions promotes cytokine and chemokine production that is a major driver of early-stage DR [6, 7, 12]. Here we also show that PGE_2_, which is produced by activated hMG, can initiate and propagate inflammatory cascades in these cells that support sustained DR progression and suggest the importance of autocrine prostanoid signaling. This finding may explain results from the subset of past clinical trials demonstrating that NSAID use slowed DR progression [18, 19]. NSAIDs, by definition, inhibit COX to reduce global prostanoid production, PGE_2_ included. Our findings are consistent with the conclusion that inhibiting COX production of proinflammatory PGE_2_ may partly or wholly explain the retinal benefits observed in these clinical studies.

While our cytokine array results revealed several cytokines and chemokines elevated by PGE_2_ stimulation, we limited our subsequent validation of cytokine levels to IL-6, IL-8, and IL-1β due to their known and well characterized roles in DR-related inflammation [31–34]. Elevation of VEGF levels by PGE_2_, an effect most relevant to PDR and angiogenesis, has been previously characterized and published by our lab in mouse Müller glia [35]; therefore, we did not reinvestigate VEGF gene expression or protein levels as readouts in this study. To date, there is limited evidence to support the roles of CXCL1 or HGF in DR pathogenesis. CXCL1, which is in the same chemokine family as IL-8, increased DR-relevant blood-retina barrier permeability in one study [43]. HGF was measured at elevated levels in the vitreous humor of PDR patients compared to nondiabetic controls in three studies and is thus hypothesized to be pro-angiogenic, yet functional consequences of this elevation are not characterized [44–46]. One study measured elevated HGF levels in the retinas of mice with STZ-induced diabetes and showed that HGF supplementation improved pericyte survival after TNFα stimulation, suggesting potential relevance of HGF in vascular permeability in DR [47]. Together, CXCL1 and HGF could also be relevant to early-stage DR despite limited studies in this experimental context. Therefore, these additional cytokines constitute reasonable targets for future investigation of the propagation of DR-relevant inflammation, particularly in Müller glia.

Further evidence of potentially distinct roles for each cytokine in the inflammatory cascade of DR pathogenesis may be found in the temporally distinct peaks of cytokine levels in this study. IL-6, IL-8, and IL-1β gene and/or protein levels were measured at maximal levels in hMG after different durations of prostaglandin stimulation: 2 or 6 h, depending on the target. The functionality of receptor antagonists to inhibit cytokine production and sustain these effects at two timepoints underscores the broad anti-inflammatory benefits that selective EP2 and/or FP receptor antagonists may provide in these cells.

Moreover, by determining that the EP2 receptor mediates proinflammatory effects of PGE_2_ in hMG, we have identified a single receptor to serve as a highly specific therapeutic target. Work from our laboratory and others has corroborated a central role of PGE_2_ signaling relevant to DR progression. One group studied the EP2 receptor as a driver of NPDR-relevant retinal endothelial cell inflammation as well as retinal vascular leakage, edema, capillary degeneration, and leukostasis in STZ-induced diabetic rats [48]. However, this group used an antagonist that is not specific for the EP2 receptor [49], prompting our further investigation to confirm that these effects are due to EP2 signaling. Furthermore, PGE_2_ signaling also has been indicated in PDR-relevant behaviors in vitro and in vivo. Our lab has demonstrated broad roles of EP4 signaling, including VEGF induction in COX-2-null mouse Müller glia, stimulation of hRMEC proliferation and tube formation, and exacerbation of the pathological response in the oxygen-induced retinopathy model use to generate PDR-like pre-retinal neovascularization [35]. Other studies have also shown that EP2 and, to a greater extent, EP3 signaling also promote angiogenesis in rat and mouse retinas [50, 51]. Collectively, these studies demonstrate a pathogenic role for PGE_2_ signaling via various EP receptors in multiple stages and pathologies of DR. The modern development of novel, highly selective EP receptor agonists and antagonists will further clarify the differences observed in past studies to advance therapeutic development.

The retinal vasculature is the primary site of DR pathology, so retinal microvascular endothelial cell dysfunction caused by treatment with diabetes-relevant stimuli can model certain aspects of DR in the cell culture setting. After observing the elevation of PGF_2α_ in hRMEC—different from PGE_2_ elevation in hMG—we concluded that these two cell types have distinct signaling mechanisms, which reflects the discrete roles of each cell type in the complex retinal architecture and in their responses to disease. PGF_2α_ has been studied in relation to DR in multiple cell types and behaviors. Interestingly, both beneficial and detrimental effects have been ascribed to PGF_2α_ signaling. In one context, PGF_2α_ signaling via the FP receptor prevented glucose-induced apoptosis of cultured human retinal pericytes, a complication indicative of early-stage DR, thereby suggesting a protective role of PGF_2α_ against DR progression [52]. In contrast, PGF_2α_-FP signaling promoted proliferation, migration, and tube formation of hRMEC as well as exacerbated retinal angiogenesis in oxygen-induced retinopathy mice, together indicating a pathological role of this prostanoid in late-stage proliferative DR [42]. Our results show that the roles of PGF_2α_ in hRMEC leukostasis endpoints, relevant to early-stage DR, align with the second study mentioned here to drive DR progression. Both the inflammatory readouts described here and the angiogenic behaviors described by Zhao et al. [42] of PGF_2α_-FP signaling in retinal endothelial cells, in contrast to the protection from apoptosis in pericytes, provide further evidence that prostanoid signaling in the retina may have complex, cell type-specific roles that demand precise targeting for therapeutic benefit.

Nonetheless, Müller glia and endothelial cells do not exist in isolation in the retina; their immediate proximity in situ suggests that their paracrine lipid signaling is important in healthy and diseased retinas. We probed the possibility of paracrine signaling by stimulating hMG and hRMEC with the prostanoid produced most highly by the opposing cell type: hMG were stimulated with PGF_2α_ and hRMEC were stimulated with PGE_2_. Here, PGF_2α_ stimulated cytokine production in hMG, and this effect was inhibited by the FP receptor antagonist AL8810. Interestingly, PGE_2_ did not stimulate adhesion molecule expression in hRMEC. These results modeling paracrine signaling show additional complexity of retinal lipid signaling, as PGF_2α_ had bioactivity in the behaviors of two retinal cell types yet PGE_2_ affected only one cell type. This further underscores the need for receptor-specific and cell type-specific therapeutic targeting of prostanoid signaling.

In testing the EP2 antagonist PF-04498148 or the FP antagonist AL8810 against stimuli modeling aspects of systemic diabetes in hMG or hRMEC, respectively, our results underscore the multifaceted disease processes that can drive inflammatory readouts relevant to DR. Prostanoid receptor antagonists yielded between 12.8% and 35.6% prevention of target gene expression in different conditions, although not all disease-relevant induction of expression could be inhibited by antagonists in the conditions tested. These partial, statistically significant effects indicate that, while the DR-relevant conditions we used here as in vitro models contribute to inflammatory propagation by multiple distinct processes, PGE_2_-EP2 and PGF_2α_-FP signaling mechanisms are important components of this complex disease.

An important limitation of our approach is the high concentrations of prostaglandin stimulus used in some experiments. Our LC–MS/MS results translate to physiologic concentrations of up to 28.2 nM for PGE_2_ in hMG and 6.71 nM for PGF_2α_ in hRMEC. In PDR patients, PGE_2_ levels were measured at 25.11 ± 11 pg/mL in the vitreous humor, compared with 16.40 ± 7 pg/mL in nondiabetic patients [40]. Similarly, 13,14-dihydro-PGF_2α_, a metabolite of PGF_2α_, was measured with a mean of 31.09 pg/mL in PDR patient vitreous humor versus 11.19 pg/mL in nondiabetic eyes[41]. Due to limitations of our cell culture models, we chose to optimize short-term (2–10 h) stimulation of retinal cell types in vitro employing relatively high concentrations of prostaglandin necessary to elicit inflammatory responses. In the diabetic patient, endogenous prostanoids are elevated at lower concentrations for years or decades over the period disease progression. It is certainly reasonable to ask if these two conditions have pathophysiological homology, but we believe that valuable information is yielded by our approach, nonetheless. As for our prostanoid receptor-focused experiments, we used prostanoid receptor antagonists at or slightly above their reported K_i_ values to avoid off-target effects and found that, even at physiologic levels, EP2 or FP receptor antagonists completely blocked stimulation by elevated PGE_2_ or PGF_2α_, respectively.

Our study was further limited to only two primary human cell types. Those we studied are of the utmost importance for DR progression: Müller glia in the homeostatic regulation of the retinal microenvironment for all cells and retinal endothelial cells in all vascular pathologies that define diabetic retinopathy. Still, the responses of all other retinal cell types to conditions of systemic diabetes may also be divergent from the PGE_2_ produced by hMG and the PGF_2α_ produced by hRMEC, and paracrine signaling effects of these two prostanoids on any other retinal cell types also remains unanswered. Future studies in retinal explants or animal models of DR would help to address the complexity of multi-cell interactions in the retina, necessary for the next steps toward therapeutic development of targeted prostanoid receptor antagonists for patients. The single cell type studies described here have yielded initial analyses of the production and molecular targets of prostanoids in early-stage DR, providing a foundation to support future translational work on this subject.

**Fig. 8:**
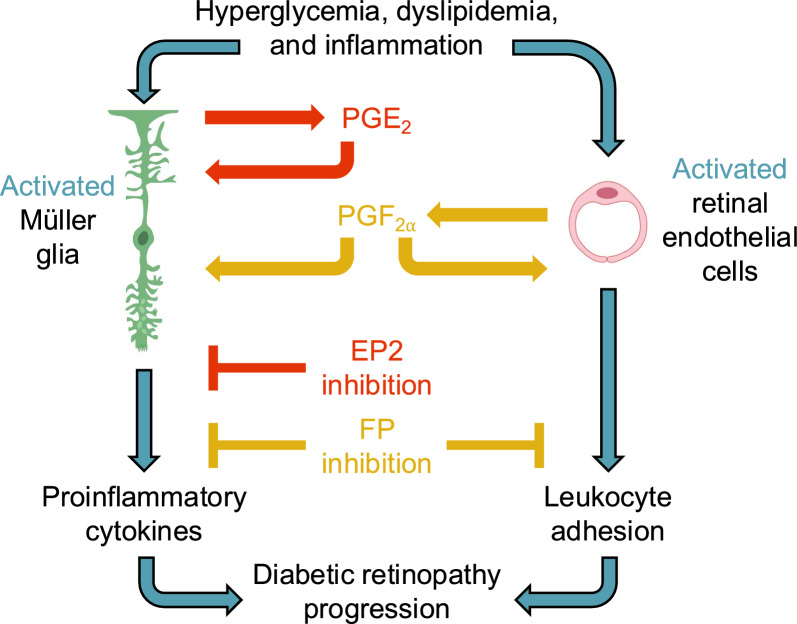
Proposed mechanisms of DR-relevant prostanoid signaling in Müller glia and retinal endothelial cells

In summary, we analyzed prostanoid signaling in two retinal cell types to identify molecular targets for inflammation relevant to early-stage DR, which currently lacks any clinical intervention. We found that primary hMG cultured in conditions modeling systemic diabetes elevate production of PGE_2_, which promotes proinflammatory cytokine production via the EP2 receptor. Additionally, primary hRMEC cultured in diabetic conditions produce PGF_2α_ most consistently, which stimulates markers of leukostasis through the FP receptor. We also modeled the putative paracrine signaling capacity of hRMEC-derived PGF_2α_ to promote cytokine production in hMG, yet PGE_2_ did not have any effect on hRMEC leukostasis markers. Our results are summarized graphically in Fig. 8. Together, our results suggest complex, cell type-specific roles for two different prostanoids and their receptors in pathologies that characterize the early inflammatory stages of DR. The mechanisms defined here will inform the future development of targeted therapies to modulate prostanoid signaling that may address NPDR without adverse side effects observed from the use of NSAIDs.

## Supplementary Information

Supplementary Material 1. A) PGE_2_ and B) PGF_2α_ measurement from hMG media after treatment with normal glucose, L-glucose, or D-glucose for 24-96 hours (n = 2–4). C) Simple linear regression of PGE_2_ production from hMG media after stimulation with palmitic acid or vehicle for 2-48 hours (n = 3–6). D) PGE_2_ measurement from hMG media after stimulation with equal 1 ng/ml concentrations of LPS, TNFα, IL-1β, or vehicle for 24 hours (n = 3). Data represent mean ± SD. Statistically significant differences are represented as *P < 0.05, **P < 0.01, ns (not significant) *P *> 0.05

Supplementary Material 2. Western blots of EP1, EP2, EP3, and EP4 receptor protein in three independent cultures of unstimulated hMG.

Supplementary Material 3. A) *IL6*, B) *CXCL8*, and C) *IL1B* gene expression in hMG after stimulation with 1 μM PGE_2_ for 2, 6, 12, or 24 hours. Data are normalized relative to DMSO vehicle-treated samples for the respective timepoints (n = 3). Data represent mean ± SD.

Supplementary Material 4. A) *CXCL8* and B) *IL1B* gene expression in hMG stimulated with vehicle or PGE_2_ ± prostanoid receptor antagonist for 2 hours (n = 3-4). C) *IL6* gene expression in hMG stimulated with vehicle or PGE_2_ ± prostanoid receptor antagonist for 6 hours (n = 3-4). D) IL-8 protein levels in culture media from hMG stimulated with vehicle or PGE_2_ ± prostanoid receptor antagonist for 6 hours (n = 3-4). E) IL-6 protein levels in culture media from hMG stimulated with vehicle or PGE_2_ ± prostanoid receptor antagonist for 10 hours (n = 3-4). Data represent mean ± SD. One-way ANOVAs with Dunnett post-hoc tests were used. Statistically significant differences are represented as *P < 0.05, **P < 0.01, ***P < 0.001, ****P < 0.0001.

Supplementary Material 5. A) *IL1B* and B) *CXCL8* gene expression in hMG stimulated with vehicle or PGF_2α_ ± FP receptor antagonist for 2 hours (n = 3). C) *IL6* gene expression in hMG stimulated with vehicle or PGF_2α_ ± FP receptor antagonist for 6 hours (n = 3). Data represent mean ± SD. One-way ANOVAs with Tukey post-hoc tests were used. Statistically significant differences are represented as *P < 0.05, **P < 0.01, ***P < 0.001, ****P < 0.0001.

Supplementary Material 6. A) *ICAM1*, B) *VCAM1*, and C) *SELE* gene expression in hRMEC stimulated with vehicle or 250 μM palmitic acid ± FP receptor antagonist for 24 hours (n = 4). D) *ICAM1*, E) *VCAM1*, and F) *SELE* gene expression in hRMEC cultured in media with normal glucose, additional 24.5 mM L-glucose, or additional 24.5 mM D-glucose ± FP receptor antagonist for 24 hours (n = 4). Data represent mean ± SD. One-way ANOVAs with Tukey post-hoc tests were used. Statistically significant differences are represented as *P < 0.05, ****P < 0.0001; ns (not significant) *P *> 0.05 shown where relevant.

## Data Availability

No datasets were generated or analysed during the current study.
