# Supplementary material for: Prostanoid signaling in retinal cells elicits inflammatory responses relevant to early-stage diabetic retinopathy
Source: J Neuroinflammation. 2024 Dec 23;21:329. doi: 10.1186/s12974-024-03319-w (PMC11667846; doi:10.1186/s12974-024-03319-w)
Supplement: Supplementary file 1 — Supplementary Material 1. A) PGE2 and B) PGF2α measurement from hMG media after treatment with normal glucose, L-glucose, or D-glucose for 24-96 hours (n = 2–4). C) Simple linear regression of PGE2 production from hMG media after stimulation with palmitic acid or vehicle for 2-48 hours (n = 3–6). D) PGE2 measurement from hMG media after stimulation with equal 1 ng/ml concentrations of LPS, TNFα, IL-1β, or vehicle for 24 hours (n = 3). Data represent mean ± SD. Statistically significant differences are represented as *P < 0.05, **P < 0.01, ns (not significant) P > 0.05 [file 12974_2024_3319_MOESM1_ESM.pdf]

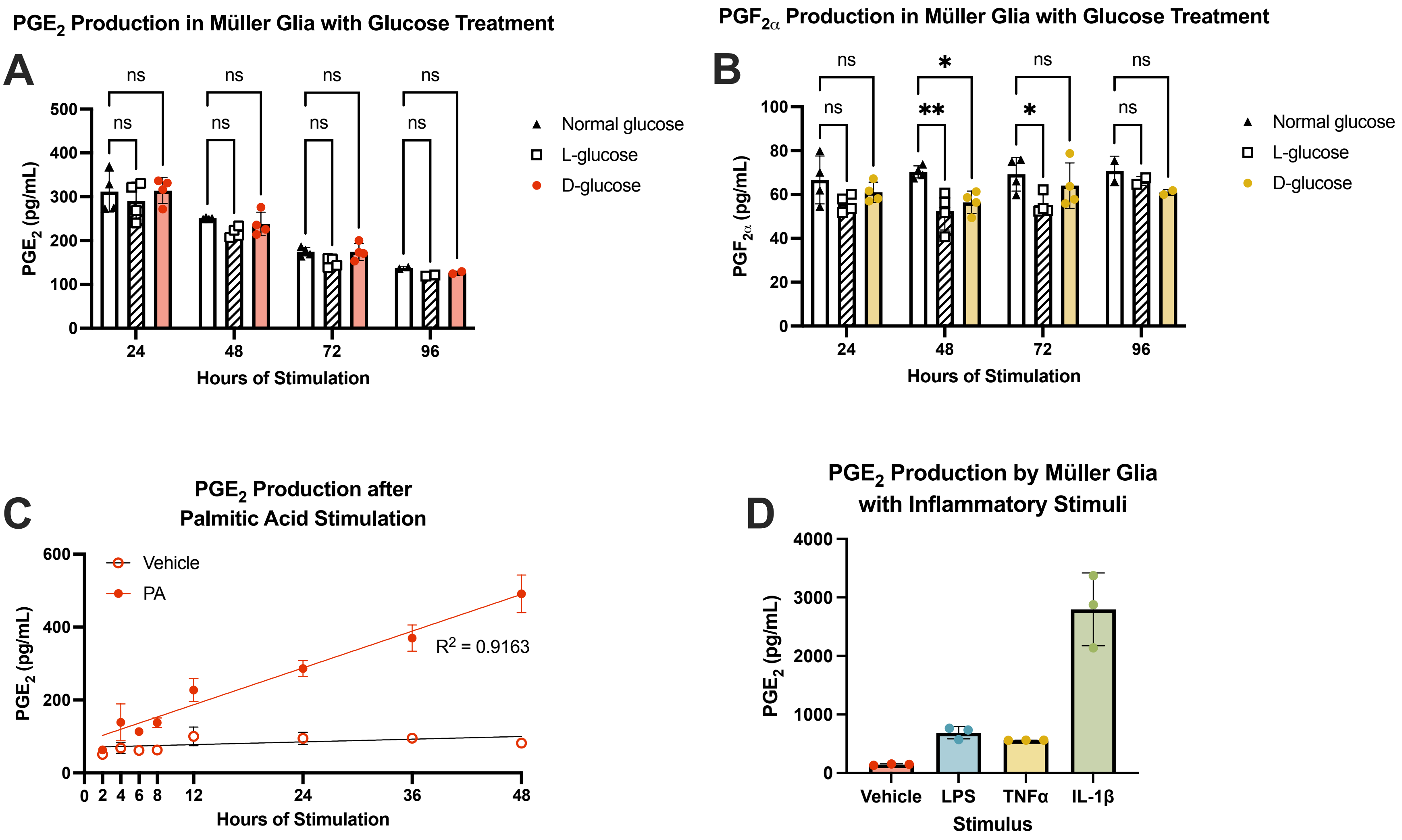

PGE2 Production in Müller Glia with Glucose Treatment
A
PGE2 (pg/mL)
Hours of Stimulation
24
48
72
96
ns
Normal glucose
L-glucose
D-glucose
PGF2α Production in Müller Glia with Glucose Treatment
B
PGF2α (pg/mL)
ns
*
**
C
PGE2 Production after Palmitic Acid Stimulation
Vehicle
PA
R2 = 0.9163
D
PGE2 Production by Müller Glia with Inflammatory Stimuli
Vehicle
LPS
TNFα
IL-1β
Stimulus
