# Supplementary figures and images for: Prostanoid signaling in retinal cells elicits inflammatory responses relevant to early-stage diabetic retinopathy
Source: J Neuroinflammation. 2024 Dec 23;21:329. doi: 10.1186/s12974-024-03319-w (PMC11667846; doi:10.1186/s12974-024-03319-w)

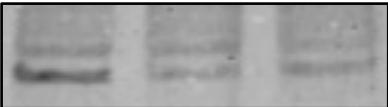

EP1

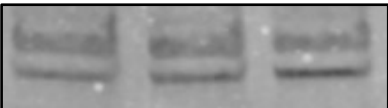

EP2

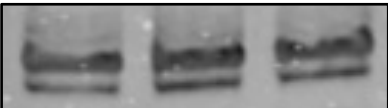

EP3

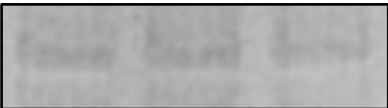

EP4

Supplement: Supplementary file 2 — Supplementary Material 2. Western blots of EP1, EP2, EP3, and EP4 receptor protein in three independent cultures of unstimulated hMG. [file 12974_2024_3319_MOESM2_ESM.pdf]

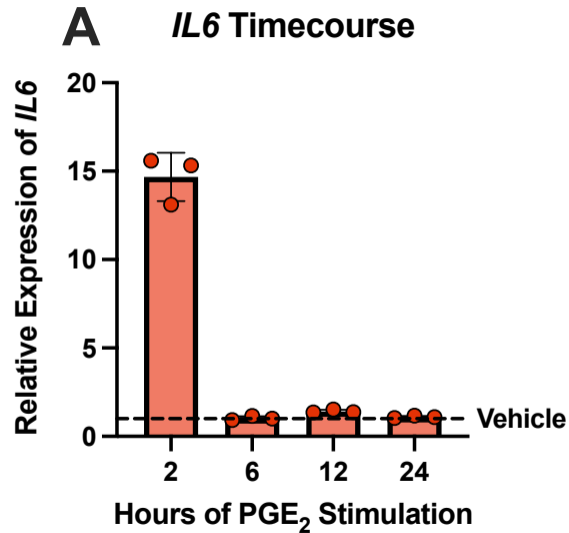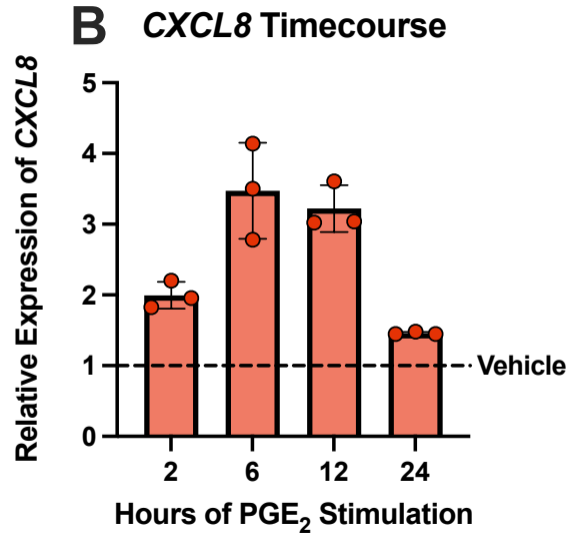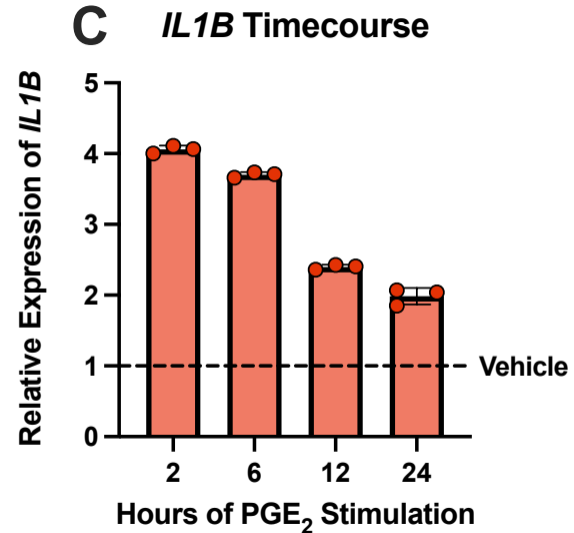

Supplement: Supplementary file 3 — Supplementary Material 3. A) IL6, B) CXCL8, and C) IL1B gene expression in hMG after stimulation with 1 μM PGE2 for 2, 6, 12, or 24 hours. Data are normalized relative to DMSO vehicle-treated samples for the respective timepoints (n = 3). Data represent mean ± SD. [file 12974_2024_3319_MOESM3_ESM.pdf]

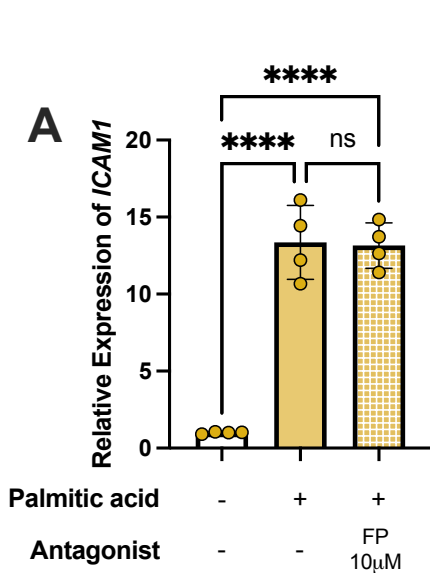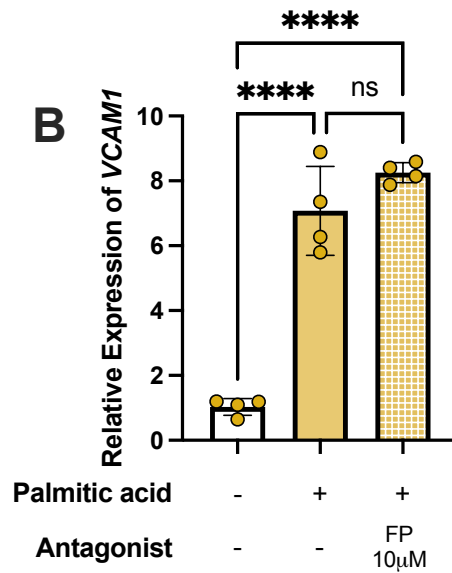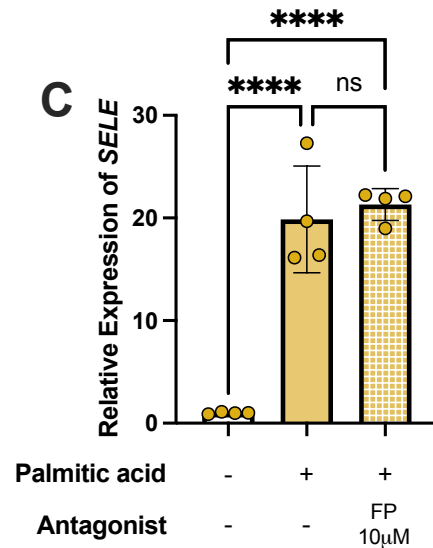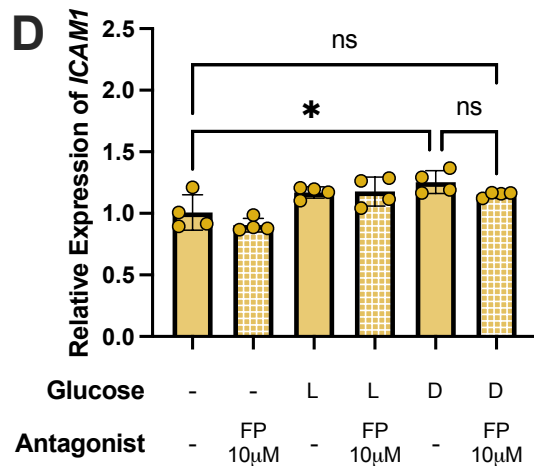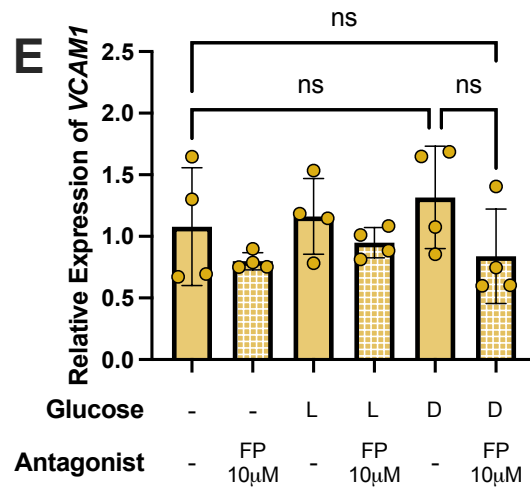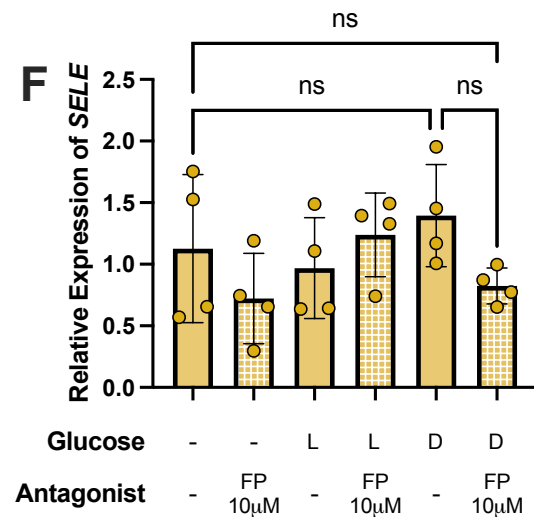

Supplement: Supplementary file 6 — Supplementary Material 6. A) ICAM1, B) VCAM1, and C) SELE gene expression in hRMEC stimulated with vehicle or 250 μM palmitic acid ± FP receptor antagonist for 24 hours (n = 4). D) ICAM1, E) VCAM1, and F) SELE gene expression in hRMEC cultured in media with normal glucose, additional 24.5 mM L-glucose, or additional 24.5 mM D-glucose ± FP receptor antagonist for 24 hours (n = 4). Data represent mean ± SD. One-way ANOVAs with Tukey post-hoc tests were used. Statistically significant differences are represented as *P < 0.05, ****P < 0.0001; ns (not significant) P > 0.05 shown where relevant. [file 12974_2024_3319_MOESM6_ESM.pdf]
