# Supplementary material for: Prostanoid signaling in retinal cells elicits inflammatory responses relevant to early-stage diabetic retinopathy
Source: J Neuroinflammation. 2024 Dec 23;21:329. doi: 10.1186/s12974-024-03319-w (PMC11667846; doi:10.1186/s12974-024-03319-w)
Supplement: Supplementary file 4 — Supplementary Material 4. A) CXCL8 and B) IL1B gene expression in hMG stimulated with vehicle or PGE2 ± prostanoid receptor antagonist for 2 hours (n = 3-4). C) IL6 gene expression in hMG stimulated with vehicle or PGE2 ± prostanoid receptor antagonist for 6 hours (n = 3-4). D) IL-8 protein levels in culture media from hMG stimulated with vehicle or PGE2 ± prostanoid receptor antagonist for 6 hours (n = 3-4). E) IL-6 protein levels in culture media from hMG stimulated with vehicle or PGE2 ± prostanoid receptor antagonist for 10 hours (n = 3-4). Data represent mean ± SD. One-way ANOVAs with Dunnett post-hoc tests were used. Statistically significant differences are represented as *P < 0.05, **P < 0.01, ***P < 0.001, ****P < 0.0001. [file 12974_2024_3319_MOESM4_ESM.pdf]

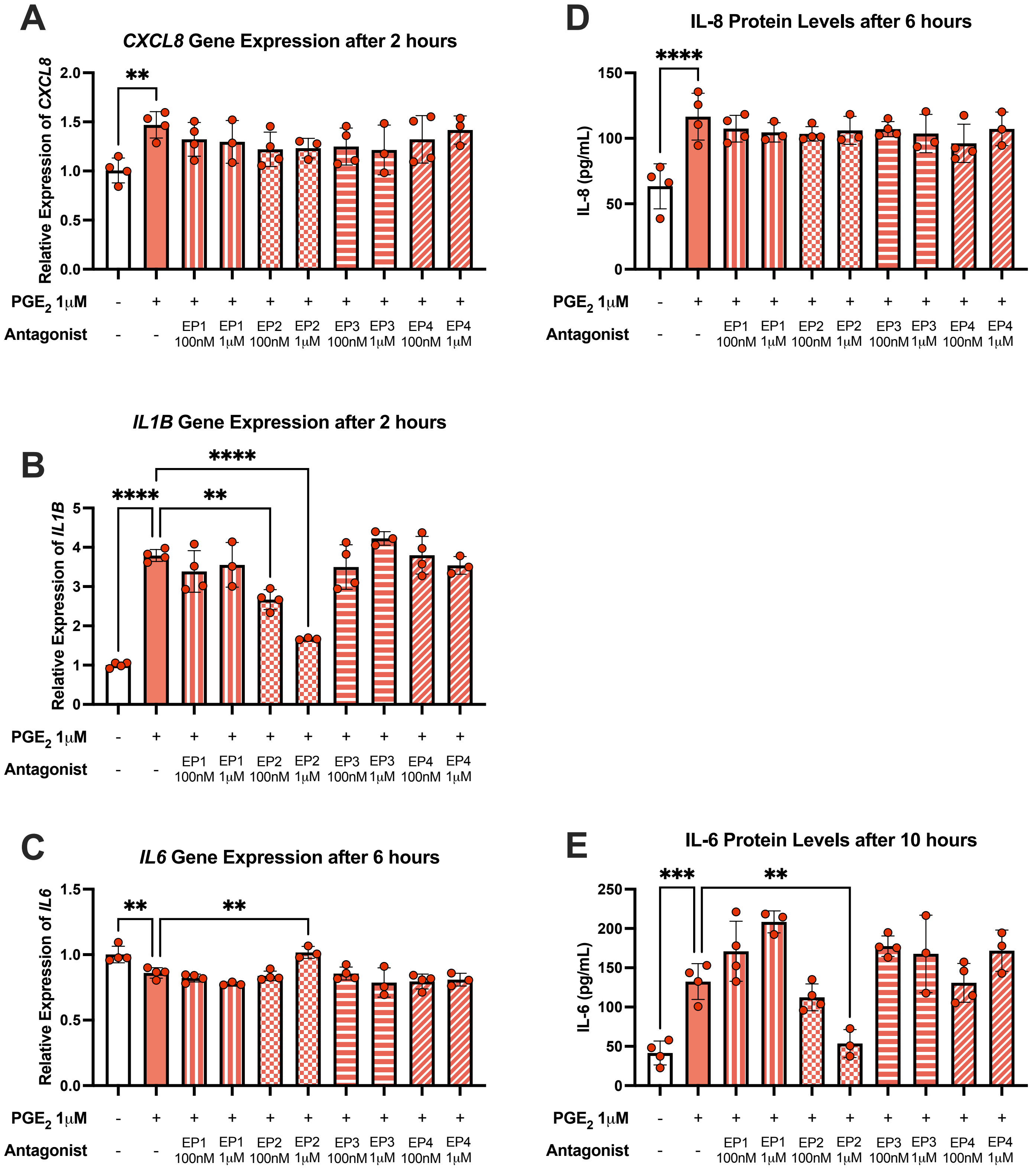
A
CXCL8 Gene Expression after 2 hours
Relative Expression of CXCL8
2.0
1.5
1.0
0.5
0.0
**
PGE2 1µM
Antagonist
EP1 100nM
EP1 1µM
EP2 100nM
EP2 1µM
EP3 100nM
EP3 1µM
EP4 100nM
EP4 1µM
B
IL1B Gene Expression after 2 hours
Relative Expression of IL1B
5
4
3
2
1
0
****
**
****
C
IL6 Gene Expression after 6 hours
Relative Expression of IL6
1.5
1.0
0.5
0.0
**
**
D
IL-8 Protein Levels after 6 hours
IL-8 (pg/mL)
150
100
50
0
****
E
IL-6 Protein Levels after 10 hours
IL-6 (pg/mL)
250
200
150
100
50
0
***
**
