# Supplementary material for: Prostanoid signaling in retinal cells elicits inflammatory responses relevant to early-stage diabetic retinopathy
Source: J Neuroinflammation. 2024 Dec 23;21:329. doi: 10.1186/s12974-024-03319-w (PMC11667846; doi:10.1186/s12974-024-03319-w)
Supplement: Supplementary file 5 — Supplementary Material 5. A) IL1B and B) CXCL8 gene expression in hMG stimulated with vehicle or PGF2α ± FP receptor antagonist for 2 hours (n = 3). C) IL6 gene expression in hMG stimulated with vehicle or PGF2α ± FP receptor antagonist for 6 hours (n = 3). Data represent mean ± SD. One-way ANOVAs with Tukey post-hoc tests were used. Statistically significant differences are represented as *P < 0.05, **P < 0.01, ***P < 0.001, ****P < 0.0001. [file 12974_2024_3319_MOESM5_ESM.pdf]

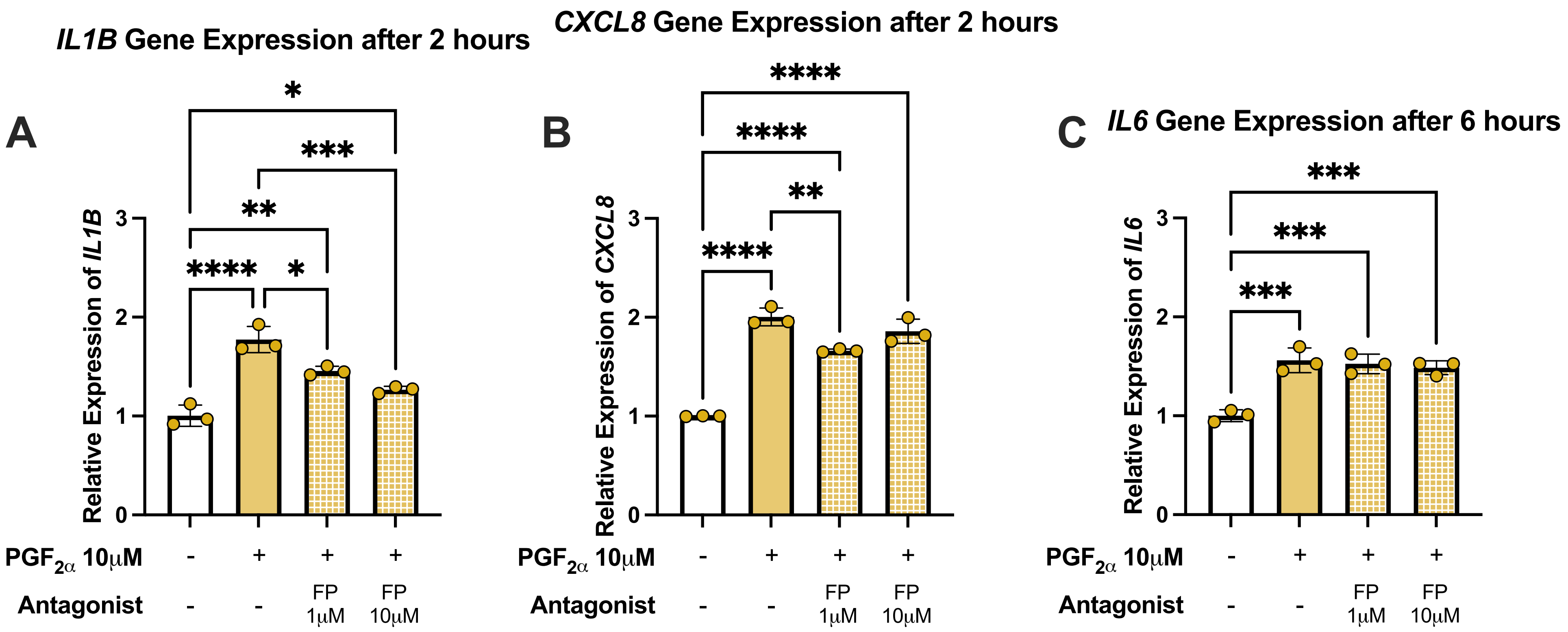
A
IL1B Gene Expression after 2 hours
Relative Expression of IL1B
B
CXCL8 Gene Expression after 2 hours
Relative Expression of CXCL8
C
IL6 Gene Expression after 6 hours
Relative Expression of IL6
PGF2α 10μM
Antagonist
FP 1μM
FP 10μM
